# Microbiome composition of *Drosophila suzukii* varies across geographical regions

**DOI:** 10.3389/fevo.2025.1696606

**Published:** 2025-12-03

**Authors:** Matthew J. Medeiros, Allexa D. Burger, Donald K. Price, Joanne Y. Yew

**Affiliations:** 1 Pacific Biosciences Research Center, School of Ocean and Earth Science and Technology, University of Hawaiʻi at Mānoa, Honolulu, HI, United States; 2 School of Life Sciences, University of Nevada at Las Vegas, Las Vegas, NV, United States

**Keywords:** *Drosophila immigrans*, Hawaiian *Drosophila*, mycobiome, fungal microbiome, microbial diversity

## Abstract

*Drosophila suzukii* is a common agricultural pest in numerous parts of the world, costing more than $500 million annually in crop loss in the United States alone. Understanding the genetic and physiological mechanisms underlying its remarkable adaptability has been a major focus for the agricultural industry as well as evolutionary biologists. The microbiome, the community of microbes associated with host organisms, can play a pivotal role in local adaptation by improving host resilience to environmental stress and providing access to new sources of nutrition. Here, we test the hypothesis that the colonization of nonnative regions is associated with the incorporation of regionally-specific microbial taxa. We compare the microbiome profiles of wild-caught *D. suzukii* across five global sites, Asia, Europe, the United Kingdom, North America, and Hawaiʻi. We also compare microbial communities of *D. suzukii* found in Hawaiʻi to another local invasive species, *D. immigrans*, and native Hawaiian drosophilids. Our results reveal that wild-caught *D. suzukii* from Asia, Europe, the United Kingdom, North America, and the Hawaiian Islands exhibit distinct microbial compositions indicating that the environment is a stronger driver of microbiome composition than species identity. Seven bacterial families were conserved between all wild *D. suzukii* populations. Within Hawaiʻi, non-native *D. suzukii* bacterial communities differed from those of native Hawaiian *Drosophila* species as well as non-native *D. immigrans*. By contrast, fungal microbiome profiles between the Hawaiian *Drosophila* and two invasive species closely resemble each other. In sum, all populations of *D. suzukii* in this study contain a subset of conserved bacterial families but also incorporate local bacterial taxa. This strategy may contribute to the rapid range expansion of *D. suzukii* and enhance its ability to exploit new dietary sources.

## Introduction

The spotted wing *Drosophila*, *Drosophila suzukii*, is considered a globally invasive pest, having spread from its native range in East Asia (Kanzawa, 1939; Bolda et al., 2010) to disparate locations in North America (Hauser, 2011), Europe (Calabria et al., 2012), South American (Deprá et al., 2014; Andreazza et al., 2017), North African countries (Kwadha et al., 2021), and Asia (Calabria et al., 2012; Cini et al., 2012). *D. suzukii’s* preference to oviposit in soft-flesh fruits has resulted in significant yield losses of fruit crops including cherries, grapes, and plums (Tait et al., 2021). The success of *D. suzukii* in expanding its range is partly attributed to its high adaptability to novel habitats and ecological niches (Poyet et al., 2015; Little et al., 2020). Within Hawaiʻi, the species is found across the islands of Kauaʻi, Oʻahu, Molokaʻi, Maui, and Hawaiʻi Island (Kaneshiro, 1983; Leblanc et al., 2009; Hauser, 2011) in lower elevation agricultural parks as well as high elevation native forest reserves. *D. suzukii* is capable of thriving in elevations over 2000 m, and at mean annual temperatures of less than 12 °C (Sánchez-Ramos et al., 2019a, b; Curbelo et al., 2022), near the lowest temperatures considered viable for activity in this genus (Koštál et al., 2016). In addition, *D. suzukii* has been observed feeding on a variety of native and non-native fruits (Magnacca et al., 2008; Koch et al., 2020). Previous genetic analyses of *D. suzukii* populations from Asia (Feng et al., 2024), Hawaiʻi (Koch et al., 2020), the continental United States (Mérel et al., 2021), Europe, and South America (Adrion et al., 2014) identified genetic divergences that may facilitate successful environmental adaptation. The microbiome has also been hypothesized as a major driver of local evolution allowing organisms to exploit new ecological niches by enhancing host physiology and behavior (Shu et al., 2021). Indeed, the ability to consume novel foods in a newly colonized area has been proposed as a key factor for successful invasion (Shik and Dussutour, 2020). *D. suzukii* uses microbes to aid metabolism and survival in their preferred high-sugar and low-protein fruit hosts (Bing et al., 2018; Gao et al., 2023) and may use naturally occurring microbes on a new host plant to extract essential nutrients (Lin et al., 2021). Local microbes may also aid in the tolerance of temperatures near the extreme of their ranges (Mueller et al., 2011; Chevalier et al., 2015; Houwenhuyse et al., 2021).

To address the possibility that the colonization of new ecological niches by *D. suzukii* is associated with changes in microbiome composition, we compared bacterial profiles of wild *D. suzukii* populations from the native ranges in China and Japan to populations from non-native ranges in Europe, the United Kingdom (UK), North America, and Hawaiʻi (Martinez-Sañudo et al., 2018) and lab-reared *D. suzukii* from the United States (US) (Bing et al., 2018; Martinez-Sañudo et al., 2018; Lin et al., 2021). To assess how closely *D. suzukii* microbiomes resembles that of other native and invasive species found in similar habitats, we compared Hawaiian *D. suzukii* bacterial and fungal profiles to another cosmopolitan drosophilid found in Hawaiʻi, *D. immigrans*, as well as to native Hawaiian picture-wing *Drosophila*.

## Materials and methods

### Sample collection of drosophilids in the Hawaiian Islands

*D. suzukii*, *D. immigrans*, and endemic Hawaiian drosophilidae were collected from the islands of Molokaʻi, Lanaʻi, and Hawaiʻi Island using sponges baited with mushrooms, banana, and yeast. Samples were immediately placed into 95% ethanol, transported on ice packs, and stored at −80 °C in the laboratory until processing. Metadata associated with samples collected in Hawaiʻi are provided in Supplementary Tables S1, S2.

### Library preparation and sequencing of Hawaiian dosophilidae

Details of DNA extraction and library preparation are as previously described (Medeiros et al., 2025). Briefly, surface-sterilized flies were homogenized using a bead mill homogenizer (Bead Ruptor Elite, Omnic, Inc; GA, USA) and DNA extracted with PowerMag Bead Solution kit (Qiagen; MD, USA) according to manufacturer’s instructions. The 16S rRNA gene was amplified with primers to the V4 region (515F: GTGYCAGCMGCCGCGGTAA; 806R: GGACTACNVGGGTWTCTAAT) (Parada et al., 2016). Fungal diversity was characterized using primers to the internal transcribed spacer (ITS1f: CTTGGTCATTTAGAGGAAGTAA; ITS2: GCTGCGTTCTTCATCGATGC) (White et al., 1990). The primers contain a 12-base pair Golay-indexed code for demultiplexing. The PCRs were performed with the KAPA3G Plant kit (Sigma Aldrich, MO, USA) using the following parameters: 95 °C for 3 min, followed by 35 cycles of 95 °C for 20 seconds, 50 °C for 15 seconds, 72 °C for 30 seconds, and a final extension for 72 °C for 3 min. The PCR products were cleaned and normalized with the Just-a-plate kit (Charm Biotech, MO, USA). High throughput sequencing (HTS) was performed with MiSeq and 250 bp paired-end kits (Illumina, Inc., CA, USA).

### Data processing of 16S rRNA amplicons for multi-region comparisons

Taxonomic analyses of *D. suzukii*, *D. immigrans*, and native Hawaiian *Drosophila* microbiomes were assessed from next-generation amplicon sequencing of regions in the 16S rRNA gene using DADA2 v1.16 (Callahan et al., 2016). All analyses were conducted using *R version 4.4.2* (R Core Team, 2022). Four publicly accessible projects deposited on the NCBI Sequence Read Archive (SRA, https://www.ncbi.nlm.nih.gov/sra) and in-house data derived from *D. suzukii*, *D. immigrans*, and native Hawaiian *Drosophila* collected in Hawaiʻi by the authors of this study were used in the analysis for a total of five independent sources of sequencing data (Supplementary Table S3). FASTQ files deposited onto the SRA were retrieved using fasterqdump command, part of the NCBI SRA Toolkit. SRA projects included in this study are PRJEB50289 (Fountain et al., 2018), PRJNA347319 (Martinez-Sañudo et al., 2018), PRJNA412893, and PRJNA719706 (Lin et al., 2021). All raw sequence reads were demultiplexed before analysis.

All sequence data were generated from paired-end Illumina sequencing strategy, however there was no consensus in the primer sets used for all projects. Additionally, two of the SRA projects (PRJNA347319 and PRJNA719706) reported paired-end read strategy but only one spot contained read information from the SRA database; thus, only one file per sample was extracted. Visualization of the quality profile plots of these data revealed that the SRA data contained merged forward and reverse reads, referred to herein as extended fragments. Due to the unique nature of each dataset used in this study, each project was handled separately for pre-processing. A custom *R* script was used to search for the presence of sequencing primers in the reads. Sequencing primers were reported for all but SRA projects PRJNA412893, and in this case the primers used were inferred using the custom script to search for common 16S rRNA sequencing primers until the primer sites were identified. Locations of the primer sites in the forward and reverse reads were used to define the trimming (trimLeft) and the quality plots were used to define the truncation (truncLen) filtering parameters in DADA2. A default max EE setting of 2 was used for both forward and reverse reads, but one project required raising the threshold to recover enough sequence reads passing the filter. For extended fragments, as suggested by the DADA2 creator on github, the error rates were inflated by 3 to account for the heterogeneity between the merged subsegments using the command inflateErr. Project-specific details including primers, and pre-processing parameters are given in Supplementary Table S4.

After filtering, trimming and estimating error rates, paired-end reads were merged following the standard DADA2 workflow. Only merged reads or extended fragments (i.e., previously merged reads) were used for downstream analysis. The merged reads (and extended fragments) for 16S rRNA gene sequences from all five projects were used to create a sequence table, remove chimeras and assign taxonomy (excluding mitochondria and chloroplasts) using the SILVA SSU Ref NR database version 138.1 (Quast et al., 2013; Yilmaz et al., 2014). The number of reads tracked through the processing pipeline for each *D. suzukii* sample is given in Supplementary Table S5. Taxonomy assignments for 16S rRNA reads were based on amplicon sequence variant (ASV) data (100% similarity). Each sample was rarefied with a subsampling depth of 5,000 ASVs.

### Data processing of ITS amplicons from Hawaiʻi drosophilidae samples

Post-processing of HTS data (filtering, trimming, and clustering) for Hawaiʻi samples (native Hawaiian flies, *D. immigrans*, *D. suzukii*) was performed using the “MetaFlow∣mics” Fungal ITS pipeline for fungi which uses the DADA2 workflow (Arisdakessian et al., 2020; Medeiros et al., 2024). ASV clustering was performed at the 97% similarity threshold. Taxonomy assignments for ITS reads were performed with NCBI BLAST, UNITE (Nilsson et al., 2019), and MycoBank (Robert et al., 2013) using a >95% sequence similarity cutoff.

### Statistical analysis

To quantify alpha-diversity, we used Chao1 and Shannon index. To assess beta-diversity, we used Bray-Curtis dissimilarity and performed ordination analyses with non-metric multidimensional scaling (NMDS). Analysis of Similarity (ANOSIM) and permutational multivariate analysis of variance (PERMANOVA) tests were applied to test for significant differences in community composition. Analyses were performed after clustering at the family or genus level and using R version 4.4.1, and the *phyloseq* package (McMurdie and Holmes, 2013). Venn diagrams were generated using complete sample sets for each population and based on the top ten families for *each* population (https://bioinformatics.psb.ugent.be/webtools/Venn/).

## Results

### Comparison of bacterial microbiomes of *D. suzukii* across native and invasive ranges and lab-maintained populations

**FIGURE 1:**
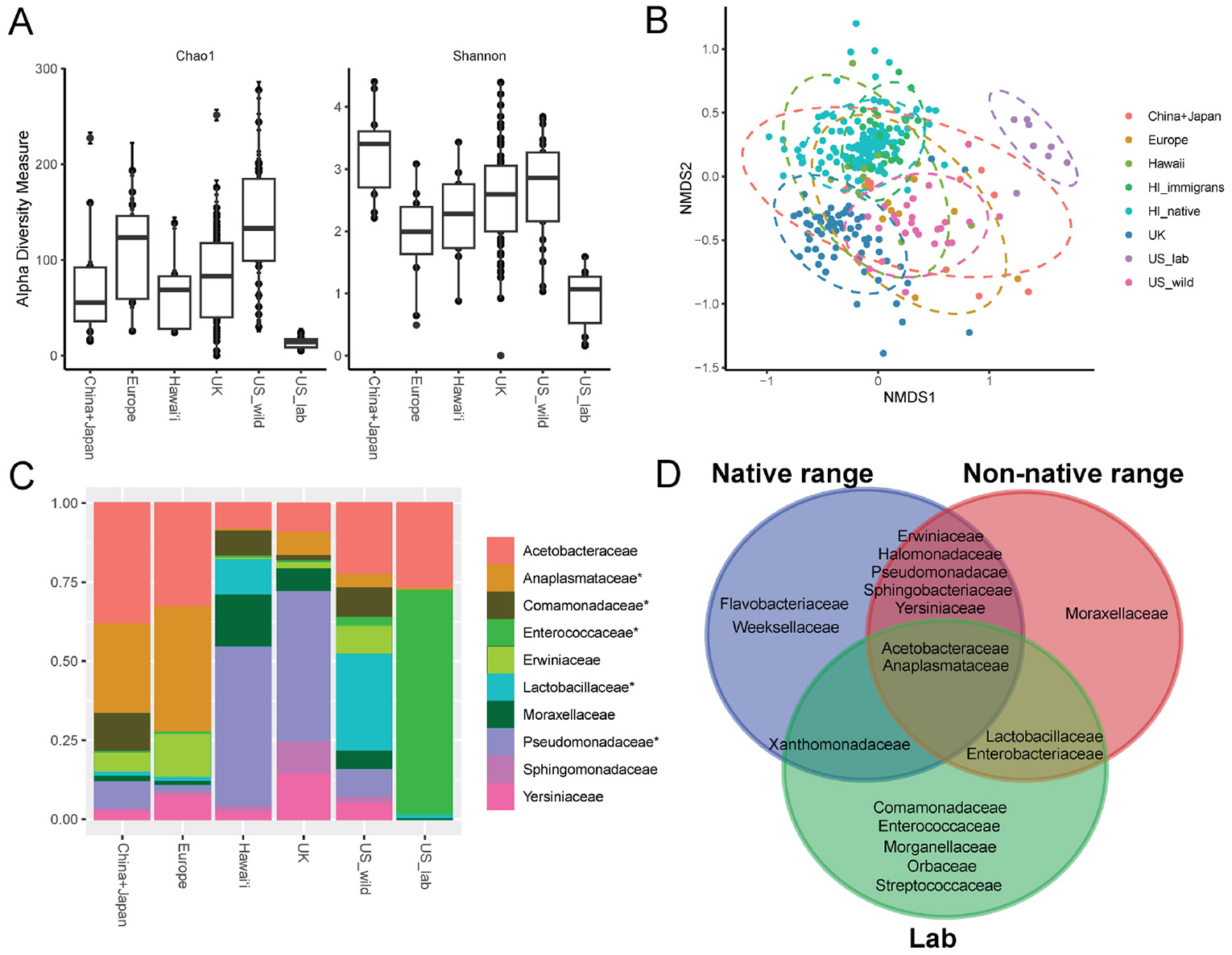
Bacterial community profiles of *D. suzukii* populations from native and non-native ranges, and lab settings. Outcomes of statistical comparisons are detailed in Tables 1, 2. (**A**) Alpha-diversity analyses based on ASVs from wild and lab populations from all sites. Each point represents a single fly. (**B**) Non-metric multidimensional scaling (NMDS) plot depicting Bray-Curtis dissimilarity distances for wild and lab populations from all sites; ANOSIM *p* = 0.001, R = 0.61. Each point represents a single fly. Populations are compared at the family level. Ellipses represent 95% confidence intervals. (**C**) Relative abundance plots of the 10 most abundant bacterial families common to all sites; *: taxa that differed significantly (f-test, *p* < 0.0001). (**D**) Venn diagram based on the 10 most abundant taxa for each population showing overlapping bacterial families; native range: n = 8, non-native range: n = 110, lab: n = 16 for all analyses.

To perform a multi-region comparison of *D. suzukii* microbiomes, we analyzed the bacterial communities of samples collected from China, Japan, North America, Europe, the UK and Hawaiʻi. In terms of alpha-diversity, populations from the native range tended to have higher compositional richness and evenness compared to Europe, UK, and Hawaiʻi when grouped by family (Figure 1A, Table 1). In addition, almost all populations of *D. suzukii* exhibit distinct compositional profiles (ANOSIM *p* = 0.001, R = 0.61; Figure 1B, Table 2). However, one exception to this general pattern is that *D. suzukii* from the native ranges of China and Japan and the non-native range of Europe exhibited similar compositions (PERMANOVA *p* = 0.075; Table 2). Flies collected in Hawaiʻi, regardless of species, clustered together in the NMDS ordination plot (Figure 1B) although *D. suzukii* from Hawaiʻi differed significantly in compositional profile from other Hawaiian populations (PERMANOVA, *p* = 0.001; Table 2). Only two bacterial families were unique to flies collected in their native range of China and Japan (Flavobacteriaceae and Weeksellaceae), suggesting that this cosmopolitan species associates with microbes beyond those that are specific to its region of origin. Seven bacterial families were common to all wild-caught *D. suzukii*, potentially serving as a core microbial community: Acetobacteraceae, Anaplasmatacea, Erwiniaceae, Halomonadaceae, Pseudomonadacae, Sphingobacteriaceae, and Yersiniaceae (Figures 1C, D). At the genus level, only the endosymbiont *Wolbachia* was common to lab and wild flies (Supplementary Figure S2, Supplementary Table S6).

The bacterial community richness of lab populations was significantly lower compared to each of the wild populations, consistent with previous studies (Chandler et al., 2011; Staubach et al., 2013) (Figure 1A, Table 1). Lab flies from US, China, and Italy contained distinct communities of bacteria, with each profile correlating with geographical location (ANOSIM *p* = 0.001 R = 0.78; Supplementary Figure S1, Supplementary Table S7). However, there were only modest differences in terms of alphadiversity (Supplementary Table S7).

### Bacterial and fungal gut microbiome comparison of native Hawaiian *Drosophila*, *D. immigrans* in Hawaiʻi, and *D. suzukii* in Hawaiʻi

**FIGURE 2:**
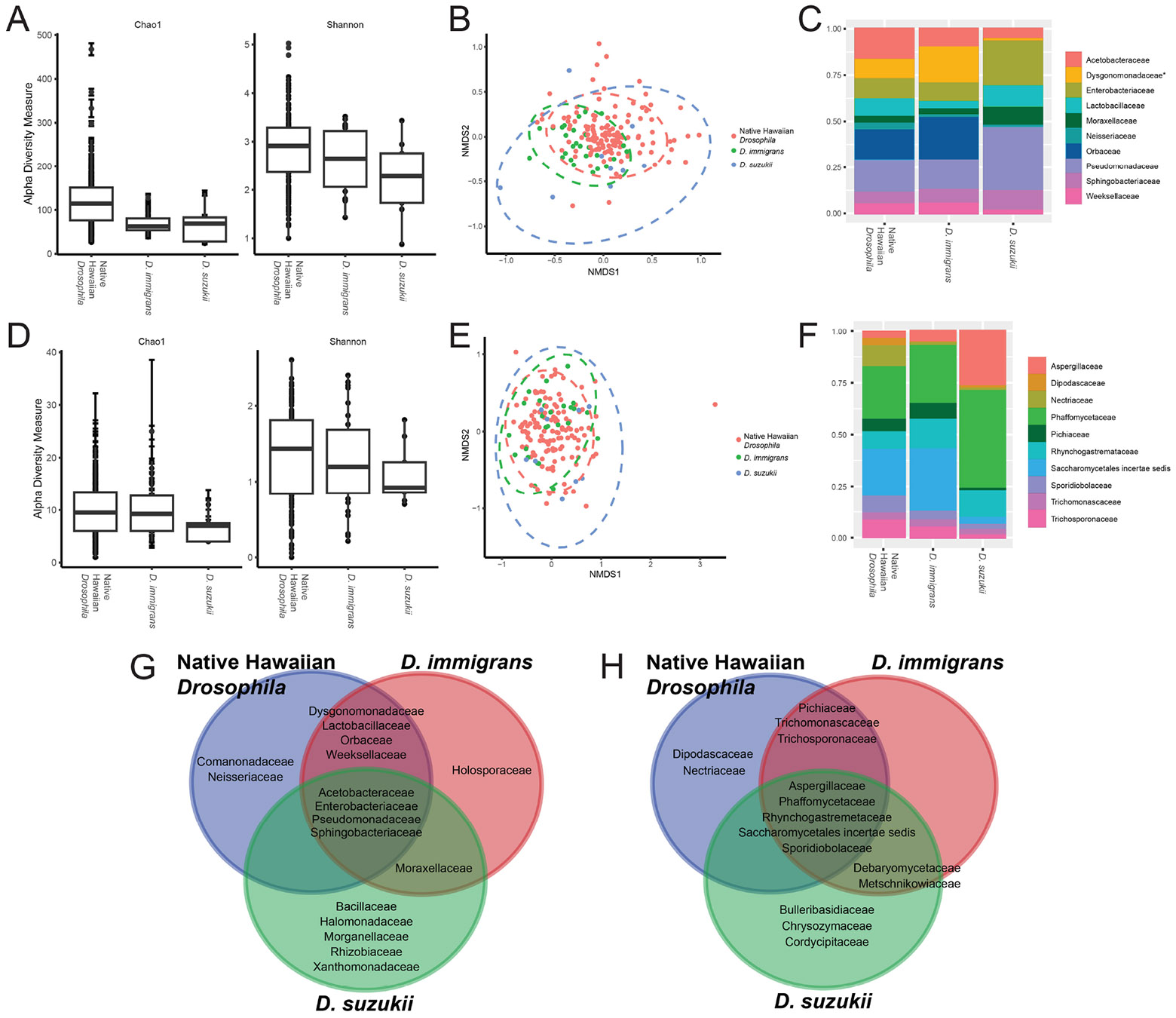
Bacterial and fungal community profiles for native Hawaiian *Drosophila*, *D. immigrans*, and *D. suzukii* flies collected from Molokaʻi, Lanaʻi, and Hawaiʻi islands. Outcomes of statistical comparisons are detailed in Table 3. (**A**) Alpha-diversity analyses based on ASVs reveal no significant differences in bacterial profiles. Each point represents a single fly. (**B**) Non-metric multidimensional scaling (NMDS) plot depicting Bray-Curtis dissimilarity distances; ANOSIM: *p* = 0.003, R = 0.18. Each point represents a single fly. Populations are compared at the family level. Ellipses represent 95% confidence intervals. (**C**) Relative abundance plots indicating the 10 most prevalent bacterial families found between the three populations; *taxa that differed significantly (f-test, *p* < 0.0001). (**D**) Alpha-diversity analyses based on ASVs reveal no significant differences in fungal profiles. (**E**) NMDS plot depicting Bray-Curtis dissimilarity distances; ANOSIM: *p* = 0.01, R = 0.12. Ellipses represent 95% confidence intervals. (**F**) Relative abundance plots showing the 10 most prevalent fungal families common to the three populations. There were no significant differences in relative abundances (f-test, *p* > 0.05). (**G**) Venn diagram based on the 10 most abundant taxa for each population showing bacterial families common to each of the Hawaiʻi populations; for all 16S rRNA analyses, native Hawaiian *Drosophila*, n = 128, *D. immigrans*, n = 27, *D. suzukii*, n = 9. (**H**) Venn diagram based on the 10 most abundant taxa for each population showing overlapping fungal families; native Hawaiian *Drosophila*; for all ITS analyses n = 129, *D. immigrans*, n = 28, *D. suzukii*, n = 9.

We predicted that *D. suzukii* colonization of new ecological niches may rely on the incorporation of local microbes. To test this possibility, we compared the bacterial and fungal profiles of *D. suzukii* to another invasive species established in Hawaiʻi, *D. immigrans*, as well as to native Hawaiian *Drosophila* found at the same sites. In terms of community richness (Chao1), both invasive species exhibited significantly lower bacterial alpha-diversity compared to native flies (Figure 2A, Table 3). However, for fungal profiles, no significant differences in alpha-diversity were found between the three populations (Figure 2D, Table 3). Additionally, *D. suzukii* contained distinct bacterial communities compared to either *D. immigrans* or native Hawaiian flies at both family (ANOSIM *p* = 0.003, R = 0.18; Figure 2G) and genus levels (ANOSIM *p* = 0.012, R = 0.15; Supplementary Figure S2, Supplementary Table S6). Five families were found in *D. suzukii* that were not common to other Hawaiian populations. Of these, Rhizobiaceae was also not detected in *D. suzukii* from other regions and may reflect enrichment from the local environment. At the genus level, *Citrobacter* and *Zymobacter* appear to be enriched only in *D. suzukii* found in Hawaiʻi and no other Hawaiian populations or regions (Supplementary Figure S2). Four bacterial families were common to invasive and native species in Hawaiʻi whereas a single family is found in both invasive species, *Moraxellaceae* (Figure 2G). The presence of the *Moraxellaxeae* family in both Hawaiʻi *D. immigrans* and *D. suzukii* as well as *D. suzukii* populations outside of its native range may indicate a conserved ecological association with invasive drosophilids.

With respect to the fungal microbiome, native Hawaiian flies and both invasive populations exhibited similar profiles in terms of taxonomic composition (ANOSIM *p* = 0.01, R = 0.117) and alpha-diversity (Figure 2). Native and invasive species share five fungal families. As with bacteria, three fungal families appear unique to *D. suzukii*: Cordycipitaceae, likely a Diptera pathogen (Naranjo-Lázaro et al., 2014), Bulleribasidiaceae, which in its yeast state is used by *D. suzukii* as a food source (Jiménez-Padilla et al., 2020), and Chrysozymaceae, a yeast previously identified from ghost moth gut (*Thitarodes* sp.) (Liu et al., 2021).

## Discussion

Microbes sourced from the local environment play myriad roles in host physiology including nutrient scavenging (Yamada et al., 2015; Bing et al., 2018), toxin inactivation (Kohl et al., 2014; Zhang et al., 2024), stress resilience (Houwenhuyse et al., 2021; Tefit et al., 2023; Price et al., 2025), lipid metabolism, and sleep regulation (Tefit et al., 2023). Given the broad range and global invasion of *D. suzukii*, we hypothesized that local microbial associations might accompany colonization and regional establishment. Specifically, we predicted that microbiomes of *D. suzukii* from different sites would contain different communities and more closely resemble those of local drosophilids. Alternatively, successful colonization could be aided by a core microbiome that is maintained regardless of host location. Our analysis of *D. suzukii* microbiomes collected from three continents and two islands provides evidence for both of these scenarios. While these results identify compositional trends rather than functional roles, they establish a valuable foundation for future experiments to test how microbiome variation influences colonization and host physiology.

### Multi-region trends and local influences on the *D. suzukii* bacterial microbiome

Wild populations from each of the major sites exhibited distinct compositional differences, indicating geographically structured variation in microbiome composition. However, three of the seven families present in all wild *D. suzukii, Acetobacteraceae, Erwiniaceae*, and *Pseudomonadaceae*, were also found in a recent survey of wild *D. suzukii* populations from Oregon (USA) and Missouri (USA) (Bhandari et al., 2025), consistent with the possibility that taxa from these families form stable, mutualistic associations with *D. suzukii*. Two of the *D. suzukii*-associated bacterial families have species members with sugar production and cellulose degradation activities, and may facilitate *D. suzukii*’s ability to use multiple host fruits (Gao et al., 2023; Netrusov et al., 2023; Wünsche and Schmid, 2023; López-Hernández et al., 2025). Both *Acetobacteraceae* and L*actobacillaceae* are considered facultative members of the *D. melanogaster* microbiome that promote host growth and participate in nutritional mutualism (Storelli et al., 2011; Pais et al., 2018). The *Erwiniaceae* family is also found in another widespread invasive insect, ambrosia beetles, for which it provides nutritional support and antibiotic protection (Cambronero-Heinrichs et al., 2023). The incorporation of bacterial taxa that offer enhanced metabolic capabilities is a common strategy observed in multiple invasive host species. Understanding the functional contributions of these microbes to *D. suzukii* adaptation may identify novel strategies for population control (Hamby and Becher, 2016).

### Comparing the microbiomes of invasive species to native Hawaiian species

Although *D. suzukii* in Hawaiʻi share features of their microbial profile with populations from other regions, their microbiome composition more closely resembles that of other Hawaiian drosophilids. This outcome indicates that *D. suzukii* populations may both retain a subset of widespread bacterial families regardless of area of occurrence, yet enrich for other groups of local bacteria when colonizing a new habitat. For example, the Rhizobiaceae family, which appears to be enriched only in Hawaiʻi *D. suzukii*, belong to the Bacteroidetes phyla, members of which are capable of degrading simple and complex polysaccharidess (Vera-Ponce de León et al., 2020; Nweze et al., 2024). At the level of genus, two taxa appear to be enriched in Hawaiʻi *D. suzukii*: *Zymobacter* and *Citrobacte*, both of which are known to confer beneficial properties to host insects. *Zymobacter* facilitates sugar fermentation and is relatively abundant in field-collected mosquitoes and stingless bees (Hegde et al., 2018; Hall et al., 2020). In addition, *Citrobacter* provides resistances to insecticide and enhances development in black soldier flies (Cheng et al., 2017; Luo et al., 2023). Incorporation of microbes that potentially enhance metabolic capabilities may allow *D. suzukii* to expand its ecological niche. These associations may reflect conserved adaptation strategies in addition to widespread and consistent opportunistic colonization. Future studies of native vs. invasive flies from other sites are needed to determine whether this is a general strategy. Additionally, measurements of host range and adaptation paired with experimental manipulation of the microbiome will directly address the hypothesis that local microbe incorporation is a necessary step for successful colonization.

*D. immigrans* is considered a member of the guild of cosmopolitan *Drosophila* species, found worldwide (Nunney, 1996). Notably, the microbiome of *D. immigrans*, first reported in Hawaiʻi in 1948 (Mainland, 1949), more closely resembles that of endemic Hawaiian flies than *D. suzukii*, a species that arrived only in 1980 (Hauser, 2011). The extended establishment time of *D. immigrans* in Hawaiʻi compared to *D. suzukii* may have allowed the former to assimilate more of the local microbes. The mycobiome communities of invasive vs. native flies in the Hawaiian Island also exhibit similar compositional profiles. Given that flies obtain much of their microbiome through diet and environment interactions rather than vertical transmission (Douglas, 2019), the general overlap in fungal composition may reflect shared habitats among the native and invasive drosophilids in Hawaiʻi (Kaneshiro, 1983; Magnacca et al., 2008; Leblanc et al., 2009).

Overall, our findings reveal both geographic differentiation and partial overlap in *D. suzukii* microbiomes across continents and the Hawaiian Islands. These patterns are consistent with a core microbiome retained across regions and with enrichment of locally prevalent microbes. These results also suggest that plasticity in the *D. suzukii* microbiome may facilitate this species’ rapid colonization of novel ecological niches. Similar to a previous study of invasive *Siganus* fish in the Mediterranean Sea (Escalas et al., 2022), we identified a shift in microbiome composition when comparing *D. suzukii* from the native range in Asia to invaded ranges. Whether compositional change provides adaptive advantages (e.g., expanding dietary resources) remains an open question. Further functional exploration of how microbes contribute to host physiology and local adaptation through, for example, common garden transplant experiments with native vs. invasive hosts may lead to the development of new microbiome methods for population control. Incorporating microbiome manipulations and metagenomic analyses into invasion ecology could also clarify whether microbes act as opportunistic colonizers or facilitators of ecological expansion.

## Supplementary Material

Supplemental Materials

The Supplementary Material for this article can be found online at: https://www.frontiersin.org/articles/10.3389/fevo.2025.1696606/full#supplementary-material

## Figures and Tables

**TABLE 1:** *P-value* outcomes of pairwise alpha-diversity comparisons at the family level amongst *D. suzukii* multi-regional populations shown in Figure 1A.

Group 1^1^	Group 2	Alpha-diversity^2^	
		Chao1	Shannon
Europe	China + Japan	0.152	**0.002**
Hawaiʻi	China + Japan	0.941	**0.021**
Hawaiʻi	Europe	0.153	0.456
UK	China + Japan	0.447	**0.021**
UK	Europe	0.152	**0.045**
UK	Hawaiʻi	0.367	**0.003**
US_wild	China + Japan	**0.014**	0.095
US_wild	Europe	0.423	**0.021**
US_wild	Hawaiʻi	**0.003**	0.172
US_wild	UK	**0.001**	0.250
US_wild	US_lab	**0.001**	**0.001**
US_lab	China + Japan	**0.001**	**0.001**
US_lab	Europe	**0.001**	**0.005**
US_lab	Hawaiʻi	**0.001**	**0.001**
US_lab	UK	**0.001**	**0.001**

1 Europe sites consist of Italy, France, Slovenia, Switzerland, and Spain; US_wild sites consist of New York and California; US_lab sites consist of New York and California.

2 Outcomes from Wilcoxon Rank Sum tests; *p*-values < 0.05 in bold.

**TABLE 2:** *P-value* outcomes of pairwise beta-diversity comparisons at the family level amongst *D. suzukii* multi-regional populations and Hawaiʻi native and non-native flies shown in Figure 1B.

Group 1^1^	Group 2	Beta-diversity^2^
Europe	China + Japan	0.075
Hawaiʻi	China + Japan	**0.013**
Hawaiʻi	Europe	**0.002**
UK	China + Japan	**0.001**
UK	Europe	**0.001**
UK	Hawaiʻi	**0.002**
US_wild	China + Japan	**0.001**
US_wild	Europe	**0.010**
US_wild	Hawaiʻi	**0.001**
US_wild	UK	**0.001**
US_wild	US_lab	**0.001**
US_lab	China + Japan	**0.001**
US_lab	Europe	**0.001**
US_lab	Hawaiʻi	**0.001**
US_lab	UK	**0.001**
HI_immigrans	China + Japan	**0.001**
HI_immigrans	Europe	**0.001**
HI_immigrans	US_wild	**0.001**
HI_immigrans	UK	**0.001**
HI_immigrans	HI_native	0.080
HI_immigrans	Hawaiʻi	**0.001**
HI_immigrans	US_lab	**0.001**
HI_native	China + Japan	**0.001**
HI_native	Europe	**0.001**
HI_native	US_wild	**0.001**
HI_native	UK	**0.001**
HI_native	Hawaiʻi	**0.001**
HI_native	US_lab	**0.001**

1 Europe: Italy, France, Slovenia, Switzerland, and Spain; US_wild sites: New York and California; US_lab sites: New York and California; HI_immigrans: *D. immgrans* caught in Hawaiʻi; HI_native: native Hawaiian *Drosophilia*.

2 Outcomes from PERMANOVA; *p*-values < 0.05 in bold.

**TABLE 3:** *P-value* outcomes of pairwise alpha- and beta-diversity comparisons of family-level bacterial (top) and fungal (bottom) community profiles from Hawaiʻi populations shown in Figure 2.

Target gene	Group 1	Group 2	Alpha-diversity^1^		Beta-diversity^2^
			Chao1	Shannon	
16S rRNA	*D. immigrans*	Hawaiian *Drosophila*	**0.001**	0.250	0.072
	*D. suzukii*	Hawaiian *Drosophila*	**0.008**	0.113	**0.001**
	*D. suzukii*	*D. immigrans*	0.858	0.250	**0.001**
ITS	*D. immigrans*	Hawaiian *Drosophila*	0.686	0.791	0.093
	*D. suzukii*	Hawaiian *Drosophila*	0.210	0.680	0.057
	*D. suzukii*	*D. immigrans*	0.339	0.680	0.145

1 Outcomes from Wilcoxon Rank Sum tests; bold values indicate statistical significance at the *p* < 0.05 level.

2 Outcomes from PERMANOVA; bold values indicate statistical significance at the *p* < 0.05 level.

## Data Availability

The datasets presented in this study can be found in online repositories. The names of the repositories and accession number(s) can be found below: https://www.ncbi.nlm.nih.gov/, PRJNA1270093 https://www.ncbi.nlm.nih.gov/, PRJEB50289 https://www.ncbi.nlm.nih.gov/, PRJNA412893 https://www.ncbi.nlm.nih.gov/, PRJNA347319 https://www.ncbi.nlm.nih.gov/, PRJNA719706.
