# Supplementary material for: Microbiome composition of Drosophila suzukii varies across geographical regions
Source: Front Ecol Evol. Author manuscript; Available in PMC 2026 Feb 24. (PMC12928383; doi:10.3389/fevo.2025.1696606)
Supplement: Supplemental Materials [file NIHMS2138295-supplement-Supplemental_Materials.pdf]

Microbiome composition of *Drosophila suzukii* varies across geographical regions

Matthew J. Medeiros[1,2], Allexa D. Burger[1], Donald K. Price[2], and Joanne Y. Yew[1]

[1]Pacific Biosciences Research Center, School of Ocean and Earth Science and Technology, University of Hawaiʻi at Mānoa
[2]School of Life Sciences, University of Nevada at Las Vegas

Supplementary Figure and Tables

Figure S1. Bacterial community profiles of lab-raised *D. suzukii.*

Figure S2. Bacterial and fungal community profiles analyzed at the genus level for *D. suzukii* populations from native and non-native ranges and *Drosophila* populations collected from Hawaiʻi.

Table S1. Summary of Hawaiʻi collection sites and samples used for 16S rRNA analysis.

Table S2. Summary of Hawaii collection sites and species from each site used for ITS analysis.

Table S3. Summary of global *D. suzukii* data sets, collection sites, and samples used for 16S rRNA analysis.

Table S4. Pre-processing parameters for five independent sequencing projects used for bacterial taxonomic profiling of *D. suzukii*, Hawaiʻi *D. immigrans*, and native Hawaiian *Drosophila.*

Table S5. Read tracking through the taxonomy analysis pipeline for all samples in the five independent sources of sequencing data used in this analysis.

Table S6. *P-value* outcomes of pairwise beta-diversity comparisons of bacterial and fungal profiles shown in **Fig. S2**.

Table S7. *P*-values for the outcomes of pairwise alpha- and beta-diversity comparisons of bacterial community profiles (16S rRNA) shown in **Fig. S1**.

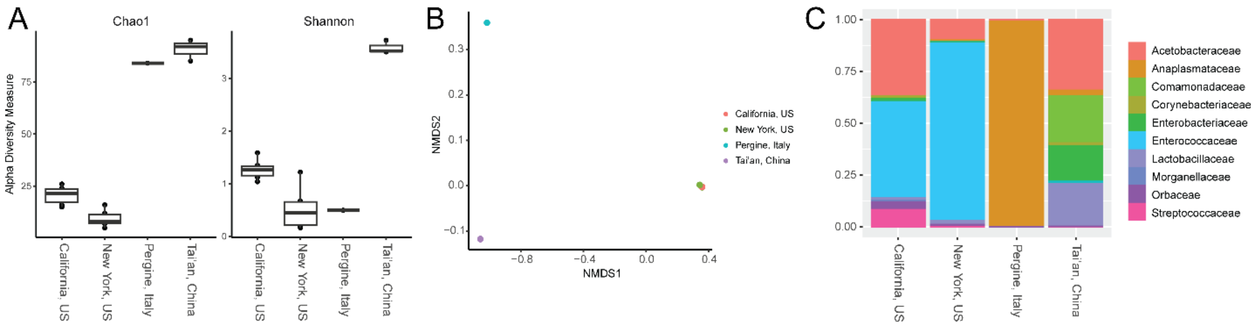

Figure S1. Bacterial community profiles of lab-raised *D. suzukii* populations. Outcomes of statistical comparisons are provided in **Table S7. (A)** Alpha-diversity analyses of lab populations from 4 labs. **(B)** Non-metric multidimensional scaling (NMDS) plot with Bray-Curtis dissimilarity distances. Populations are compared at the family level; ANOSIM $p = 0.001$, $R = 0.78$. **(C)** Relative abundance plots indicating the 10 most abundant bacterial families for all sites. No significant differences were detected between the sites (f-test, $p > 0.05$); for all analyses, n = 8 flies per population.

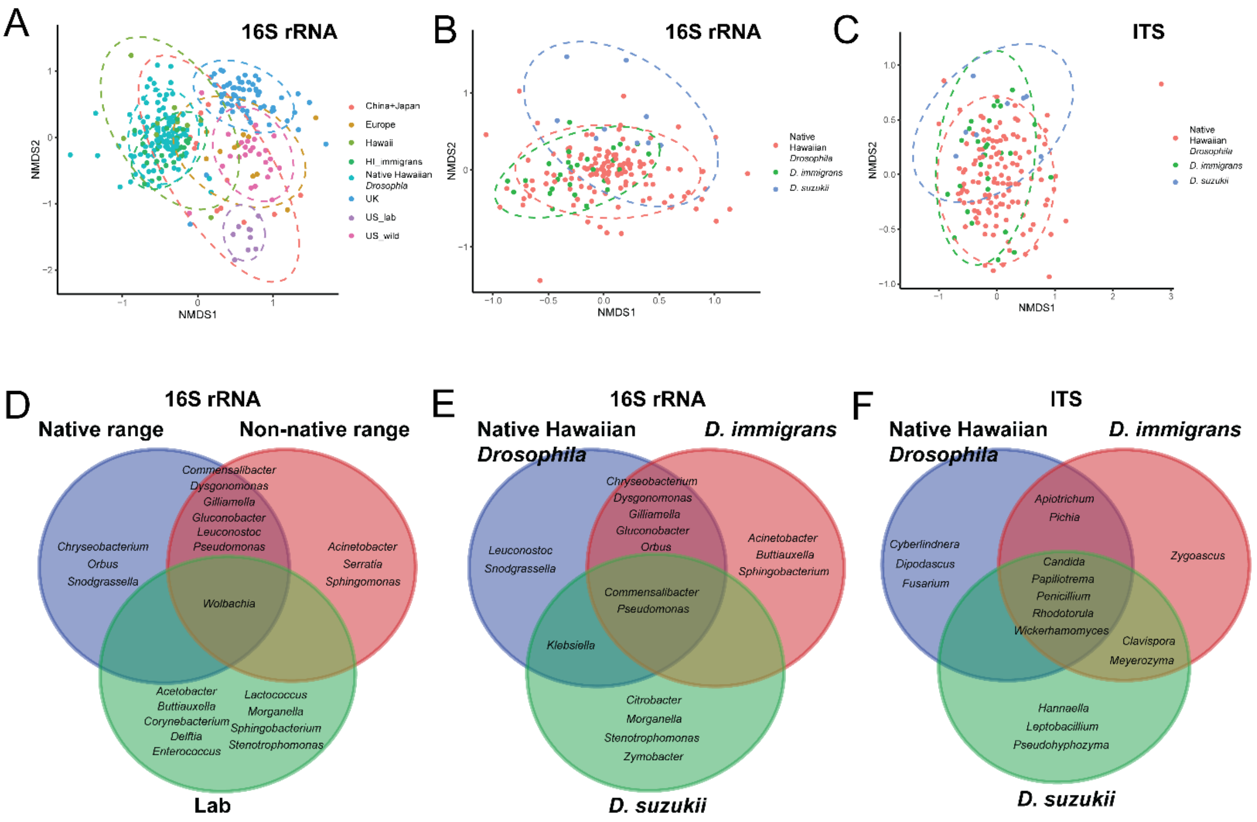

Figure S2. Bacterial and fungal community profiles analyzed at the genus level for *D. suzukii* populations from native and non-native ranges and *Drosophila* populations collected from Hawai'i. Outcomes of statistical comparisons are provided in **Table S6**. Non-metric multidimensional scaling (NMDS) plots display Bray-Curtis dissimilarity distances, with each point representing a single fly. Ellipses represent 95% confidence intervals. Venn diagrams are based on the 10 most abundant taxa for each population.

(A) NMDS plot of showing bacterial beta-diversity of wild and lab populations from all sites; ANOSIM p = 0.001, R = 0.69; native range: n = 8, non-native range: n = 110, lab: n = 16.
(B) NMDS plot of showing bacterial beta-diversity of Hawai'i populations; ANOSIM p = 0.012, R = 0.15; native Hawaiian *Drosophila,* n = 128, *D. immigrans*, n = 27, *D. suzukii*, n = 9.
(C) NMDS plot showing fungal beta-diversity of Hawai'i populations; ANOSIM p = 0.048, R = 0.08; native Hawaiian *Drosophila,* n = 129, *D. immigrans*, n = 27, *D. suzukii*, n = 9.
(D) Venn diagram comparing the bacterial profiles of *D. suzukii* from native and non-native ranges, and lab populations.
(E) Venn diagram comparing the bacterial profiles of *D. suzukii, D. immigrans*, and native Hawaiian *Drosophila* collected from Hawai'i.
(F) Venn diagram comparing the fungal profiles of *D. suzukii, D. immigrans*, and native Hawaiian *Drosophila* collected from Hawai'i.

Table S1. Summary of Hawaiʻi collection sites and samples used for 16S rRNA analysis.

Name	Project	Source	Country	Locality_1	Locality_2[†]	Year	Month	Status	Species identification	Latitude	Longitude
16S_538_S39_L001	PRJNA1270093	UHM (this study)	United States	Hawaiʻi	Mo_Hanalilolilo	2021	5	Native	Drosophila_cilifera	21.126	-156.915
16S_517_S18_L001	PRJNA1270093	UHM (this study)	United States	Hawaiʻi	Mo_PuuKolekole	2021	5	Native	Drosophila_grimshawi	21.118	-156.908
16S_534_S35_L001	PRJNA1270093	UHM (this study)	United States	Hawaiʻi	Mo_Hanalilolilo	2021	5	Native	Drosophila_bostrycha	21.126	-156.915
16S_537_S38_L001	PRJNA1270093	UHM (this study)	United States	Hawaiʻi	Mo_Hanalilolilo	2021	5	Native	Drosophila_bostrycha	21.126	-156.915
16S_500_S1_L001	PRJNA1270093	UHM (this study)	United States	Hawaiʻi	Mo_PuuKolekole	2021	5	Native	Drosophila_fasciculisetae	21.118	-156.908
16S_501_S2_L001	PRJNA1270093	UHM (this study)	United States	Hawaiʻi	Mo_PuuKolekole	2021	5	Native	Drosophila_fasciculisetae	21.118	-156.908
16S_502_S3_L001	PRJNA1270093	UHM (this study)	United States	Hawaiʻi	Mo_PuuKolekole	2021	5	Native	Drosophila_fasciculisetae	21.118	-156.908
16S_503_S4_L001	PRJNA1270093	UHM (this study)	United States	Hawaiʻi	Mo_PuuKolekole	2021	5	Native	Drosophila_fasciculisetae	21.118	-156.908
16S_504_S5_L001	PRJNA1270093	UHM (this study)	United States	Hawaiʻi	Mo_PuuKolekole	2021	5	Native	Drosophila_fasciculisetae	21.118	-156.908
16S_505_S6_L001	PRJNA1270093	UHM (this study)	United States	Hawaiʻi	Mo_PuuKolekole	2021	5	Native	Drosophila_fasciculisetae	21.118	-156.908
16S_506_S7_L001	PRJNA1270093	UHM (this study)	United States	Hawaiʻi	Mo_PuuKolekole	2021	5	Native	Drosophila_fasciculisetae	21.118	-156.908
16S_507_S8_L001	PRJNA1270093	UHM (this study)	United States	Hawaiʻi	Mo_PuuKolekole	2021	5	Native	Drosophila_fasciculisetae	21.118	-156.908
16S_508_S9_L001	PRJNA1270093	UHM (this study)	United States	Hawaiʻi	Mo_PuuKolekole	2021	5	Native	Drosophila_fasciculisetae	21.118	-156.908
16S_509_S10_L001	PRJNA1270093	UHM (this study)	United States	Hawaiʻi	Mo_PuuKolekole	2021	5	Native	Drosophila_fasciculisetae	21.118	-156.908
16S_510_S11_L001	PRJNA1270093	UHM (this study)	United States	Hawaiʻi	Mo_PuuKolekole	2021	5	Native	Drosophila_fasciculisetae	21.118	-156.908
16S_511_S12_L001	PRJNA1270093	UHM (this study)	United States	Hawaiʻi	Mo_PuuKolekole	2021	5	Native	Drosophila_fasciculisetae	21.118	-156.908
16S_512_S13_L001	PRJNA1270093	UHM (this study)	United States	Hawaiʻi	Mo_PuuKolekole	2021	5	Native	Drosophila_fasciculisetae	21.118	-156.908
16S_530_S31_L001	PRJNA1270093	UHM (this study)	United States	Hawaiʻi	Mo_Pepeopae	2021	5	Native	Drosophila_fasciculisetae	21.118	-156.909
16S_531_S32_L001	PRJNA1270093	UHM (this study)	United States	Hawaiʻi	Mo_Pepeopae	2021	5	Native	Drosophila_fasciculisetae	21.118	-156.909
16S_539_S40_L001	PRJNA1270093	UHM (this study)	United States	Hawaiʻi	Mo_Hanalilolilo	2021	5	Native	Drosophila_fasciculisetae	21.126	-156.915
16S_540_S41_L001	PRJNA1270093	UHM (this study)	United States	Hawaiʻi	Mo_Hanalilolilo	2021	5	Native	Drosophila_fasciculisetae	21.126	-156.915
16S_541_S42_L001	PRJNA1270093	UHM (this study)	United States	Hawaiʻi	Mo_Hanalilolilo	2021	5	Native	Drosophila_fasciculisetae	21.126	-156.915
16S_542_S43_L001	PRJNA1270093	UHM (this study)	United States	Hawaiʻi	Mo_Hanalilolilo	2021	5	Native	Drosophila_fasciculisetae	21.126	-156.915
16S_543_S44_L001	PRJNA1270093	UHM (this study)	United States	Hawaiʻi	Mo_Hanalilolilo	2021	5	Native	Drosophila_fasciculisetae	21.126	-156.915
16S_513_S14_L001	PRJNA1270093	UHM (this study)	United States	Hawaiʻi	Mo_PuuKolekole	2021	5	Native	Drosophila_neoperkinsi	21.118	-156.908
16S_514_S15_L001	PRJNA1270093	UHM (this study)	United States	Hawaiʻi	Mo_PuuKolekole	2021	5	Native	Drosophila_neoperkinsi	21.118	-156.908
16S_515_S16_L001	PRJNA1270093	UHM (this study)	United States	Hawaiʻi	Mo_PuuKolekole	2021	5	Native	Drosophila_neoperkinsi	21.118	-156.908
16S_516_S17_L001	PRJNA1270093	UHM (this study)	United States	Hawaiʻi	Mo_PuuKolekole	2021	5	Native	Drosophila_neoperkinsi	21.118	-156.908
16S_535_S36_L001	PRJNA1270093	UHM (this study)	United States	Hawaiʻi	Mo_Hanalilolilo	2021	5	Native	Drosophila_neoperkinsi	21.126	-156.915
16S_536_S37_L001	PRJNA1270093	UHM (this study)	United States	Hawaiʻi	Mo_Hanalilolilo	2021	5	Native	Drosophila_neoperkinsi	21.126	-156.915
16S_522_S23_L001	PRJNA1270093	UHM (this study)	United States	Hawaiʻi	Mo_PuuKolekole	2021	5	Non-native	Drosophila_immigrans	21.118	-156.908
16S_524_S25_L001	PRJNA1270093	UHM (this study)	United States	Hawaiʻi	Mo_PuuKolekole	2021	5	Non-native	Drosophila_immigrans	21.118	-156.908
16S_545_S46_L001	PRJNA1270093	UHM (this study)	United States	Hawaiʻi	Mo_Hanalilolilo	2021	5	Non-native	Drosophila_immigrans	21.126	-156.915
16S_546_S47_L001	PRJNA1270093	UHM (this study)	United States	Hawaiʻi	Mo_Hanalilolilo	2021	5	Non-native	Drosophila_immigrans	21.126	-156.915
16S_523_S24_L001	PRJNA1270093	UHM (this study)	United States	Hawaiʻi	Mo_PuuKolekole	2021	5	Non-native	Drosophila_suzukii	21.118	-156.908
16S_558_S59_L001	PRJNA1270093	UHM (this study)	United States	Hawaiʻi	Mo_Hanalilolilo	2021	5	Non-native	Drosophila_suzukii	21.126	-156.915
16S_645_S149_L001	PRJNA1270093	UHM (this study)	United States	Hawaiʻi	L_Puhielelu_exclosure	2021	7	Native	Drosophila_grimshawi	20.806	-156.862
16S_647_S151_L001	PRJNA1270093	UHM (this study)	United States	Hawaiʻi	L_Puhielelu_exclosure	2021	7	Non-native	Drosophila_immigrans	20.806	-156.862
16S_648_S152_L001	PRJNA1270093	UHM (this study)	United States	Hawaiʻi	L_Puhielelu_exclosure	2021	7	Non-native	Drosophila_immigrans	20.806	-156.862
16S_649_S153_L001	PRJNA1270093	UHM (this study)	United States	Hawaiʻi	L_Puhielelu_exclosure	2021	7	Non-native	Drosophila_immigrans	20.806	-156.862
16S_651_S155_L001	PRJNA1270093	UHM (this study)	United States	Hawaiʻi	L_Puhielelu_parking	2021	7	Non-native	Drosophila_immigrans	20.806	-156.865
16S_653_S157_L001	PRJNA1270093	UHM (this study)	United States	Hawaiʻi	L_Lanaihale	2021	7	Non-native	Drosophila_immigrans	20.813	-156.874
16S_654_S158_L001	PRJNA1270093	UHM (this study)	United States	Hawaiʻi	L_Lanaihale	2021	7	Non-native	Drosophila_immigrans	20.813	-156.874
16S_656_S160_L001	PRJNA1270093	UHM (this study)	United States	Hawaiʻi	L_Lanaihale	2021	7	Non-native	Drosophila_immigrans	20.813	-156.874
16S_652_S156_L001	PRJNA1270093	UHM (this study)	United States	Hawaiʻi	L_Puhielelu_parking	2021	7	Non-native	Drosophila_suzukii	20.806	-156.865
16S_657_S161_L001	PRJNA1270093	UHM (this study)	United States	Hawaiʻi	L_Lanaihale	2021	7	Non-native	Drosophila_suzukii	20.813	-156.874
16S_658_S162_L001	PRJNA1270093	UHM (this study)	United States	Hawaiʻi	L_Lanaihale	2021	7	Non-native	Drosophila_suzukii	20.813	-156.874
16S_659_S163_L001	PRJNA1270093	UHM (this study)	United States	Hawaiʻi	L_Lanaihale	2021	7	Non-native	Drosophila_suzukii	20.813	-156.874

Name	Project	Source	Country	Locality_1	Locality_2[†]	Year	Month	Status	Species identification	Latitude	Longitude
16S_571_S72_L001	PRJNA1270093	UHM (this study)	United States	Hawai'i	H_Olaa_Pole44	2021	3	Native	Drosophila_setosimentum	19.462	-155.248
16S_619_S123_L001	PRJNA1270093	UHM (this study)	United States	Hawai'i	H_Puu_Makaala	2021	3	Native	Drosophila_setosimentum	19.484	-155.271
16S_620_S124_L001	PRJNA1270093	UHM (this study)	United States	Hawai'i	H_Puu_Makaala	2021	3	Native	Drosophila_setosimentum	19.484	-155.271
16S_621_S125_L001	PRJNA1270093	UHM (this study)	United States	Hawai'i	H_Puu_Makaala	2021	3	Native	Drosophila_setosimentum	19.484	-155.271
16S_335_S77_L001	PRJNA1270093	UHM (this study)	United States	Hawai'i	H_Saddle_Rd	2020	10	Native	Drosophila_medialis	19.675	-155.332
16S_10_S8_L001	PRJNA1270093	UHM (this study)	United States	Hawai'i	H_Toms_Trail	2020	10	Native	Drosophila_ochracea	19.574	-155.216
16S_116_S64_L001	PRJNA1270093	UHM (this study)	United States	Hawai'i	H_Toms_Trail	2020	10	Native	Drosophila_ochracea	19.574	-155.216
16S_120_S66_L001	PRJNA1270093	UHM (this study)	United States	Hawai'i	H_Toms_Trail	2020	10	Native	Drosophila_sproati	19.574	-155.216
16S_153_S76_L001	PRJNA1270093	UHM (this study)	United States	Hawai'i	H_Toms_Trail	2020	10	Native	Drosophila_sproati	19.574	-155.216
16S_154_S77_L001	PRJNA1270093	UHM (this study)	United States	Hawai'i	H_Toms_Trail	2020	10	Native	Drosophila_ochracea	19.574	-155.216
16S_156_S79_L001	PRJNA1270093	UHM (this study)	United States	Hawai'i	H_Toms_Trail	2020	10	Native	Drosophila_sproati	19.574	-155.216
16S_27_S18_L001	PRJNA1270093	UHM (this study)	United States	Hawai'i	H_Toms_Trail	2020	10	Native	Drosophila_sproati	19.574	-155.216
16S_28_S19_L001	PRJNA1270093	UHM (this study)	United States	Hawai'i	H_Toms_Trail	2020	10	Native	Drosophila_sproati	19.574	-155.216
16S_29_S20_L001	PRJNA1270093	UHM (this study)	United States	Hawai'i	H_Toms_Trail	2020	10	Native	Drosophila_sproati	19.574	-155.216
16S_319_S61_L001	PRJNA1270093	UHM (this study)	United States	Hawai'i	H_Army_Rd	2020	12	Native	Drosophila_sproati	19.568	-155.230
16S_32_S22_L001	PRJNA1270093	UHM (this study)	United States	Hawai'i	H_HETF	2019	5	Native	Drosophila_sproati	19.912	-155.313
16S_322_S64_L001	PRJNA1270093	UHM (this study)	United States	Hawai'i	H_Army_Rd	2020	12	Native	Drosophila_sproati	19.568	-155.230
16S_323_S65_L001	PRJNA1270093	UHM (this study)	United States	Hawai'i	H_Army_Rd	2020	12	Native	Drosophila_sproati	19.568	-155.230
16S_33_S23_L001	PRJNA1270093	UHM (this study)	United States	Hawai'i	H_HETF	2019	5	Native	Drosophila_sproati	19.912	-155.313
16S_332_S74_L001	PRJNA1270093	UHM (this study)	United States	Hawai'i	H_Saddle_Rd	2020	12	Native	Drosophila_sproati	19.675	-155.332
16S_34_S24_L001	PRJNA1270093	UHM (this study)	United States	Hawai'i	H_HETF	2019	5	Native	Drosophila_sproati	19.912	-155.313
16S_35_S25_L001	PRJNA1270093	UHM (this study)	United States	Hawai'i	H_HETF	2019	5	Native	Drosophila_murphyi	19.912	-155.313
16S_36_S26_L001	PRJNA1270093	UHM (this study)	United States	Hawai'i	H_HETF	2019	5	Native	Drosophila_sproati	19.912	-155.313
16S_37_S27_L001	PRJNA1270093	UHM (this study)	United States	Hawai'i	H_HETF	2019	5	Native	Drosophila_sproati	19.912	-155.313
16S_38_S28_L001	PRJNA1270093	UHM (this study)	United States	Hawai'i	H_Toms_Trail	2020	10	Native	Drosophila_ochracea	19.574	-155.216
16S_380_S234_L001	PRJNA1270093	UHM (this study)	United States	Hawai'i	H_KauFR	2021	3	Native	Drosophila_sproati	19.341	-155.460
16S_381_S235_L001	PRJNA1270093	UHM (this study)	United States	Hawai'i	H_KauFR	2021	3	Native	Drosophila_sproati	19.341	-155.460
16S_382_S236_L001	PRJNA1270093	UHM (this study)	United States	Hawai'i	H_KauFR	2021	3	Native	Drosophila_sproati	19.341	-155.460
16S_383_S237_L001	PRJNA1270093	UHM (this study)	United States	Hawai'i	H_KauFR	2021	3	Native	Drosophila_murphyi	19.341	-155.460
16S_384_S238_L001	PRJNA1270093	UHM (this study)	United States	Hawai'i	H_KauFR	2021	3	Native	Drosophila_murphyi	19.341	-155.460
16S_385_S239_L001	PRJNA1270093	UHM (this study)	United States	Hawai'i	H_KauFR	2021	3	Native	Drosophila_murphyi	19.341	-155.460
16S_399_S254_L001	PRJNA1270093	UHM (this study)	United States	Hawai'i	H_KauFR	2021	3	Native	Drosophila_murphyi	19.341	-155.460
16S_400_S255_L001	PRJNA1270093	UHM (this study)	United States	Hawai'i	H_KauFR	2021	3	Native	Drosophila_murphyi	19.341	-155.460
16S_401_S256_L001	PRJNA1270093	UHM (this study)	United States	Hawai'i	H_KauFR	2021	3	Native	Drosophila_murphyi	19.341	-155.460
16S_402_S257_L001	PRJNA1270093	UHM (this study)	United States	Hawai'i	H_KauFR	2021	3	Native	Drosophila_murphyi	19.341	-155.460
16S_403_S258_L001	PRJNA1270093	UHM (this study)	United States	Hawai'i	H_KauFR	2021	3	Native	Drosophila_murphyi	19.341	-155.460
16S_404_S259_L001	PRJNA1270093	UHM (this study)	United States	Hawai'i	H_KauFR	2021	3	Native	Drosophila_sproati	19.341	-155.460
16S_406_S261_L001	PRJNA1270093	UHM (this study)	United States	Hawai'i	H_KauFR	2021	3	Native	Drosophila_sproati	19.341	-155.460
16S_407_S262_L001	PRJNA1270093	UHM (this study)	United States	Hawai'i	H_KauFR	2021	3	Native	Drosophila_sproati	19.341	-155.460
16S_567_S68_L001	PRJNA1270093	UHM (this study)	United States	Hawai'i	H_Olaa_Pole44	2021	3	Native	Drosophila_sproati	19.462	-155.248
16S_568_S69_L001	PRJNA1270093	UHM (this study)	United States	Hawai'i	H_Olaa_Pole44	2021	3	Native	Drosophila_sproati	19.462	-155.248
16S_569_S70_L001	PRJNA1270093	UHM (this study)	United States	Hawai'i	H_Olaa_Pole44	2021	3	Native	Drosophila_sproati	19.462	-155.248
16S_570_S71_L001	PRJNA1270093	UHM (this study)	United States	Hawai'i	H_Olaa_Pole44	2021	3	Native	Drosophila_sproati	19.462	-155.248
16S_596_S100_L001	PRJNA1270093	UHM (this study)	United States	Hawai'i	H_Olaa_Pole44	2021	3	Native	Drosophila_sproati	19.462	-155.248
16S_597_S101_L001	PRJNA1270093	UHM (this study)	United States	Hawai'i	H_Olaa_Pole44	2021	3	Native	Drosophila_sproati	19.462	-155.248
16S_598_S102_L001	PRJNA1270093	UHM (this study)	United States	Hawai'i	H_Olaa_Pole44	2021	3	Native	Drosophila_sproati	19.462	-155.248
16S_599_S103_L001	PRJNA1270093	UHM (this study)	United States	Hawai'i	H_Olaa_Pole44	2021	3	Native	Drosophila_sproati	19.462	-155.248
16S_611_S115_L001	PRJNA1270093	UHM (this study)	United States	Hawai'i	H_Puu_Makaala	2021	3	Native	Drosophila_sproati	19.484	-155.271
16S_612_S116_L001	PRJNA1270093	UHM (this study)	United States	Hawai'i	H_Puu_Makaala	2021	3	Native	Drosophila_sproati	19.484	-155.271
16S_613_S117_L001	PRJNA1270093	UHM (this study)	United States	Hawai'i	H_Puu_Makaala	2021	3	Native	Drosophila_sproati	19.484	-155.271

Name	Project	Source	Country	Locality_1	Locality_2[†]	Year	Month	Status	Species identification	Latitude	Longitude
16S_614_S118_L001	PRJNA1270093	UHM (this study)	United States	Hawai'i	H_Puu_Makaala	2021	3	Native	Drosophila_sproati	19.484	-155.271
16S_615_S119_L001	PRJNA1270093	UHM (this study)	United States	Hawai'i	H_Puu_Makaala	2021	3	Native	Drosophila_sproati	19.484	-155.271
16S_616_S120_L001	PRJNA1270093	UHM (this study)	United States	Hawai'i	H_Puu_Makaala	2021	3	Native	Drosophila_sproati	19.484	-155.271
16S_617_S121_L001	PRJNA1270093	UHM (this study)	United States	Hawai'i	H_Puu_Makaala	2021	3	Native	Drosophila_sproati	19.484	-155.271
16S_643_S147_L001	PRJNA1270093	UHM (this study)	United States	Hawai'i	H_Olaa_Pole44	2021	3	Native	Drosophila_ochracea	19.462	-155.248
16S_644_S148_L001	PRJNA1270093	UHM (this study)	United States	Hawai'i	H_Olaa_Pole44	2021	3	Native	Drosophila_ochracea	19.462	-155.248
16S_71_S49_L001	PRJNA1270093	UHM (this study)	United States	Hawai'i	H_Toms_Trail	2020	10	Native	Drosophila_ochracea	19.574	-155.216
16S_77_S51_L001	PRJNA1270093	UHM (this study)	United States	Hawai'i	H_Toms_Trail	2020	10	Native	Drosophila_ochracea	19.574	-155.216
16S_78_S52_L001	PRJNA1270093	UHM (this study)	United States	Hawai'i	H_Toms_Trail	2020	10	Native	Drosophila_ochracea	19.574	-155.216
16S_8_S6_L001	PRJNA1270093	UHM (this study)	United States	Hawai'i	H_Toms_Trail	2020	10	Native	Drosophila_ochracea	19.574	-155.216
16S_80_S53_L001	PRJNA1270093	UHM (this study)	United States	Hawai'i	H_Toms_Trail	2020	10	Native	Drosophila_ochracea	19.574	-155.216
16S_81_S54_L001	PRJNA1270093	UHM (this study)	United States	Hawai'i	H_Toms_Trail	2020	10	Native	Drosophila_ochracea	19.574	-155.216
16S_85_S55_L001	PRJNA1270093	UHM (this study)	United States	Hawai'i	H_Toms_Trail	2020	10	Native	Drosophila_sproati	19.574	-155.216
16S_9_S7_L001	PRJNA1270093	UHM (this study)	United States	Hawai'i	H_Toms_Trail	2020	10	Native	Drosophila_ochracea	19.574	-155.216
187_S176_L001	PRJNA1270093	UHM (this study)	United States	Hawai'i	H_Toms_Trail	2020	10	Native	Drosophila_ciliaticrus	19.574	-155.216
194_S184_L001	PRJNA1270093	UHM (this study)	United States	Hawai'i	H_Toms_Trail	2020	10	Native	Drosophila_sproati	19.574	-155.216
206_S196_L001	PRJNA1270093	UHM (this study)	United States	Hawai'i	H_Saddle_Rd	2020	12	Native	Drosophila_sproati	19.675	-155.332
207_S197_L001	PRJNA1270093	UHM (this study)	United States	Hawai'i	H_Saddle_Rd	2020	12	Native	Drosophila_sproati	19.675	-155.332
16S_40_S29_L001	PRJNA1270093	UHM (this study)	United States	Hawai'i	H_Toms_Trail	2020	12	Native	Drosophila_sproati	19.574	-155.216
16S_405_S260_L001	PRJNA1270093	UHM (this study)	United States	Hawai'i	H_KauFR	2021	3	Native	Drosophila_sproati	19.341	-155.460
205_S195_L001	PRJNA1270093	UHM (this study)	United States	Hawai'i	H_Saddle_Rd	2020	12	Native	Drosophila_sproati	19.675	-155.332
16S_100_S62_L001	PRJNA1270093	UHM (this study)	United States	Hawai'i	H_Toms_Trail	2020	12	Native	Drosophila_basisetae	19.574	-155.216
16S_101_S63_L001	PRJNA1270093	UHM (this study)	United States	Hawai'i	H_Toms_Trail	2020	12	Native	Drosophila_basisetae	19.574	-155.216
16S_118_S65_L001	PRJNA1270093	UHM (this study)	United States	Hawai'i	H_Toms_Trail	2020	12	Native	Drosophila_basisetae	19.574	-155.216
16S_122_S67_L001	PRJNA1270093	UHM (this study)	United States	Hawai'i	H_Toms_Trail	2020	12	Native	Drosophila_basisetae	19.574	-155.216
16S_134_S68_L001	PRJNA1270093	UHM (this study)	United States	Hawai'i	H_Toms_Trail	2020	12	Native	Drosophila_basisetae	19.574	-155.216
16S_135_S69_L001	PRJNA1270093	UHM (this study)	United States	Hawai'i	H_Toms_Trail	2020	12	Native	Drosophila_basisetae	19.574	-155.216
16S_137_S70_L001	PRJNA1270093	UHM (this study)	United States	Hawai'i	H_Toms_Trail	2020	12	Native	Drosophila_basisetae	19.574	-155.216
16S_138_S71_L001	PRJNA1270093	UHM (this study)	United States	Hawai'i	H_Toms_Trail	2020	12	Native	Drosophila_basisetae	19.574	-155.216
16S_139_S72_L001	PRJNA1270093	UHM (this study)	United States	Hawai'i	H_Toms_Trail	2020	12	Native	Drosophila_basisetae	19.574	-155.216
16S_143_S73_L001	PRJNA1270093	UHM (this study)	United States	Hawai'i	H_Toms_Trail	2020	12	Native	Drosophila_basisetae	19.574	-155.216
16S_144_S74_L001	PRJNA1270093	UHM (this study)	United States	Hawai'i	H_Toms_Trail	2020	12	Native	Drosophila_basisetae	19.574	-155.216
16S_148_S75_L001	PRJNA1270093	UHM (this study)	United States	Hawai'i	H_Toms_Trail	2020	12	Native	Drosophila_basisetae	19.574	-155.216
16S_18_S12_L001	PRJNA1270093	UHM (this study)	United States	Hawai'i	H_Toms_Trail	2020	12	Native	Drosophila_basisetae	19.574	-155.216
16S_19_S13_L001	PRJNA1270093	UHM (this study)	United States	Hawai'i	H_Toms_Trail	2020	12	Native	Drosophila_basisetae	19.574	-155.216
16S_324_S66_L001	PRJNA1270093	UHM (this study)	United States	Hawai'i	H_Army_Rd	2020	12	Native	Drosophila_basisetae	19.568	-155.230
16S_600_S104_L001	PRJNA1270093	UHM (this study)	United States	Hawai'i	H_Olaa_Pole44	2021	3	Native	Drosophila_prolaticilia	19.462	-155.248
16S_601_S105_L001	PRJNA1270093	UHM (this study)	United States	Hawai'i	H_Olaa_Pole44	2021	3	Native	Drosophila_basisetae	19.462	-155.248
16S_602_S106_L001	PRJNA1270093	UHM (this study)	United States	Hawai'i	H_Olaa_Pole44	2021	3	Native	Drosophila_basisetae	19.462	-155.248
16S_603_S107_L001	PRJNA1270093	UHM (this study)	United States	Hawai'i	H_Olaa_Pole44	2021	3	Native	Drosophila_basisetae	19.462	-155.248
16S_604_S108_L001	PRJNA1270093	UHM (this study)	United States	Hawai'i	H_Olaa_Pole44	2021	3	Native	Drosophila_basisetae	19.462	-155.248
16S_74_S50_L001	PRJNA1270093	UHM (this study)	United States	Hawai'i	H_Toms_Trail	2020	12	Native	Drosophila_basisetae	19.574	-155.216
16S_88_S56_L001	PRJNA1270093	UHM (this study)	United States	Hawai'i	H_Toms_Trail	2020	12	Native	Drosophila_basisetae	19.574	-155.216
16S_89_S57_L001	PRJNA1270093	UHM (this study)	United States	Hawai'i	H_Toms_Trail	2020	12	Native	Drosophila_basisetae	19.574	-155.216
16S_93_S58_L001	PRJNA1270093	UHM (this study)	United States	Hawai'i	H_Toms_Trail	2020	12	Native	Drosophila_basisetae	19.574	-155.216
186_S175_L001	PRJNA1270093	UHM (this study)	United States	Hawai'i	H_Toms_Trail	2020	12	Native	Drosophila_basisetae	19.574	-155.216
195_S185_L001	PRJNA1270093	UHM (this study)	United States	Hawai'i	H_Toms_Trail	2020	12	Native	Drosophila_prolaticilia	19.574	-155.216
16S_318_S60_L001	PRJNA1270093	UHM (this study)	United States	Hawai'i	H_Army_Rd	2020	12	Native	Drosophila_basisetae	19.568	-155.230
16S_321_S63_L001	PRJNA1270093	UHM (this study)	United States	Hawai'i	H_Army_Rd	2020	12	Non-native	Drosophila_immigrans	19.568	-155.230
16S_330_S72_L001	PRJNA1270093	UHM (this study)	United States	Hawai'i	H_Saddle_Rd	2020	12	Non-native	Drosophila_immigrans	19.675	-155.332

Name	Project	Source	Country	Locality_1	Locality_2[†]	Year	Month	Status	Species identification	Latitude	Longitude
16S_393_S248_L001	PRJNA1270093	UHM (this study)	United States	Hawai'i	H_KauFR	2021	3	Non-native	Drosophila_immigrans	19.341	-155.460
16S_395_S250_L001	PRJNA1270093	UHM (this study)	United States	Hawai'i	H_KauFR	2021	3	Non-native	Drosophila_immigrans	19.341	-155.460
16S_396_S251_L001	PRJNA1270093	UHM (this study)	United States	Hawai'i	H_KauFR	2021	3	Non-native	Drosophila_immigrans	19.341	-155.460
16S_408_S263_L001	PRJNA1270093	UHM (this study)	United States	Hawai'i	H_KauFR	2021	3	Non-native	Drosophila_immigrans	19.341	-155.460
16S_412_S267_L001	PRJNA1270093	UHM (this study)	United States	Hawai'i	H_KauFR	2021	3	Non-native	Drosophila_immigrans	19.341	-155.460
16S_581_S82_L001	PRJNA1270093	UHM (this study)	United States	Hawai'i	H_Olaa_Pole44	2021	3	Non-native	Drosophila_immigrans	19.462	-155.248
16S_583_S84_L001	PRJNA1270093	UHM (this study)	United States	Hawai'i	H_Olaa_Pole44	2021	3	Non-native	Drosophila_immigrans	19.462	-155.248
16S_584_S85_L001	PRJNA1270093	UHM (this study)	United States	Hawai'i	H_Olaa_Pole44	2021	3	Non-native	Drosophila_immigrans	19.462	-155.248
16S_592_S93_L001	PRJNA1270093	UHM (this study)	United States	Hawai'i	H_Olaa_Pole44	2021	3	Non-native	Drosophila_immigrans	19.462	-155.248
16S_606_S110_L001	PRJNA1270093	UHM (this study)	United States	Hawai'i	H_Puu_Makaala	2021	3	Non-native	Drosophila_immigrans	19.484	-155.271
16S_607_S111_L001	PRJNA1270093	UHM (this study)	United States	Hawai'i	H_Puu_Makaala	2021	3	Non-native	Drosophila_immigrans	19.484	-155.271
16S_608_S112_L001	PRJNA1270093	UHM (this study)	United States	Hawai'i	H_Puu_Makaala	2021	3	Non-native	Drosophila_immigrans	19.484	-155.271
16S_610_S114_L001	PRJNA1270093	UHM (this study)	United States	Hawai'i	H_Puu_Makaala	2021	3	Non-native	Drosophila_immigrans	19.484	-155.271
16S_394_S249_L001	PRJNA1270093	UHM (this study)	United States	Hawai'i	H_KauFR	2021	3	Non-native	Drosophila_immigrans	19.341	-155.460
16S_629_S133_L001	PRJNA1270093	UHM (this study)	United States	Hawai'i	H_Kipuka_Ki	2021	3	Non-native	Drosophila_suzukii	19.442	-155.316
16S_631_S135_L001	PRJNA1270093	UHM (this study)	United States	Hawai'i	H_Kipuka_Ki	2021	3	Non-native	Drosophila_suzukii	19.442	-155.316
16S_632_S136_L001	PRJNA1270093	UHM (this study)	United States	Hawai'i	H_Kipuka_Ki	2021	3	Non-native	Drosophila_suzukii	19.442	-155.316

[†] Mo: Molokai; H: Hawaii Island; L: Lanai

Table S2. Summary of Hawai'i collection sites and samples used for ITS analysis.

Sample	Project	Source	Country	Locality_1	Locality_2[†]	Year	Month	Status	Species identification	Latitude	Longitude
186_S175_L001	PRJNA1270093	UHM (this study)	United States	Hawai'i	H_Toms_Trail	2020	12	Native	Drosophila_basisetae	19.574	-155.216
187_S176_L001	PRJNA1270093	UHM (this study)	United States	Hawai'i	H_Toms_Trail	2020	10	Native	Drosophila_ciliaticrus	19.574	-155.216
194_S184_L001	PRJNA1270093	UHM (this study)	United States	Hawai'i	H_Toms_Trail	2020	10	Native	Drosophila_sproati	19.574	-155.216
195_S185_L001	PRJNA1270093	UHM (this study)	United States	Hawai'i	H_Toms_Trail	2020	12	Native	Drosophila_prolaticilia	19.574	-155.216
205_S195_L001	PRJNA1270093	UHM (this study)	United States	Hawai'i	H_Saddle_Rd	2020	12	Native	Drosophila_sproati	19.675	-155.332
206_S196_L001	PRJNA1270093	UHM (this study)	United States	Hawai'i	H_Saddle_Rd	2020	12	Native	Drosophila_sproati	19.675	-155.332
207_S197_L001	PRJNA1270093	UHM (this study)	United States	Hawai'i	H_Saddle_Rd	2020	12	Native	Drosophila_sproati	19.675	-155.332
ITS-10_S104_L001	PRJNA1270093	UHM (this study)	United States	Hawai'i	H_Toms_Trail	2020	10	Native	Drosophila_ochracea	19.574	-155.216
ITS-100_S158_L001	PRJNA1270093	UHM (this study)	United States	Hawai'i	H_Toms_Trail	2020	12	Native	Drosophila_basisetae	19.574	-155.216
ITS-101_S159_L001	PRJNA1270093	UHM (this study)	United States	Hawai'i	H_Toms_Trail	2020	12	Native	Drosophila_basisetae	19.574	-155.216
ITS-116_S160_L001	PRJNA1270093	UHM (this study)	United States	Hawai'i	H_Toms_Trail	2020	10	Native	Drosophila_ochracea	19.574	-155.216
ITS-118_S161_L001	PRJNA1270093	UHM (this study)	United States	Hawai'i	H_Toms_Trail	2020	12	Native	Drosophila_basisetae	19.574	-155.216
ITS-120_S162_L001	PRJNA1270093	UHM (this study)	United States	Hawai'i	H_Toms_Trail	2020	10	Native	Drosophila_sproati	19.574	-155.216
ITS-122_S163_L001	PRJNA1270093	UHM (this study)	United States	Hawai'i	H_Toms_Trail	2020	12	Native	Drosophila_basisetae	19.574	-155.216
ITS-134_S164_L001	PRJNA1270093	UHM (this study)	United States	Hawai'i	H_Toms_Trail	2020	12	Native	Drosophila_basisetae	19.574	-155.216
ITS-135_S165_L001	PRJNA1270093	UHM (this study)	United States	Hawai'i	H_Toms_Trail	2020	12	Native	Drosophila_basisetae	19.574	-155.216
ITS-137_S166_L001	PRJNA1270093	UHM (this study)	United States	Hawai'i	H_Toms_Trail	2020	12	Native	Drosophila_basisetae	19.574	-155.216
ITS-138_S167_L001	PRJNA1270093	UHM (this study)	United States	Hawai'i	H_Toms_Trail	2020	12	Native	Drosophila_basisetae	19.574	-155.216
ITS-139_S168_L001	PRJNA1270093	UHM (this study)	United States	Hawai'i	H_Toms_Trail	2020	12	Native	Drosophila_basisetae	19.574	-155.216
ITS-143_S169_L001	PRJNA1270093	UHM (this study)	United States	Hawai'i	H_Toms_Trail	2020	12	Native	Drosophila_basisetae	19.574	-155.216
ITS-144_S170_L001	PRJNA1270093	UHM (this study)	United States	Hawai'i	H_Toms_Trail	2020	12	Native	Drosophila_basisetae	19.574	-155.216
ITS-148_S171_L001	PRJNA1270093	UHM (this study)	United States	Hawai'i	H_Toms_Trail	2020	12	Native	Drosophila_basisetae	19.574	-155.216
ITS-153_S172_L001	PRJNA1270093	UHM (this study)	United States	Hawai'i	H_Toms_Trail	2020	10	Native	Drosophila_sproati	19.574	-155.216
ITS-154_S173_L001	PRJNA1270093	UHM (this study)	United States	Hawai'i	H_Toms_Trail	2020	10	Native	Drosophila_ochracea	19.574	-155.216
ITS-156_S175_L001	PRJNA1270093	UHM (this study)	United States	Hawai'i	H_Toms_Trail	2020	10	Native	Drosophila_sproati	19.574	-155.216
ITS-18_S108_L001	PRJNA1270093	UHM (this study)	United States	Hawai'i	H_Toms_Trail	2020	12	Native	Drosophila_basisetae	19.574	-155.216
ITS-19_S109_L001	PRJNA1270093	UHM (this study)	United States	Hawai'i	H_Toms_Trail	2020	12	Native	Drosophila_basisetae	19.574	-155.216
ITS-27_S114_L001	PRJNA1270093	UHM (this study)	United States	Hawai'i	H_Toms_Trail	2020	10	Native	Drosophila_sproati	19.574	-155.216
ITS-28_S115_L001	PRJNA1270093	UHM (this study)	United States	Hawai'i	H_Toms_Trail	2020	10	Native	Drosophila_sproati	19.574	-155.216
ITS-29_S116_L001	PRJNA1270093	UHM (this study)	United States	Hawai'i	H_Toms_Trail	2020	10	Native	Drosophila_sproati	19.574	-155.216
ITS-318_S60_L001	PRJNA1270093	UHM (this study)	United States	Hawai'i	H_Army_Rd	2020	12	Native	Drosophila_basisetae	19.568	-155.230
ITS-319_S61_L001	PRJNA1270093	UHM (this study)	United States	Hawai'i	H_Army_Rd	2020	12	Native	Drosophila_sproati	19.568	-155.230
ITS-32_S118_L001	PRJNA1270093	UHM (this study)	United States	Hawai'i	H_HETF	2019	5	Native	Drosophila_sproati	19.912	-155.313
ITS-321_S63_L001	PRJNA1270093	UHM (this study)	United States	Hawai'i	H_Army_Rd	2020	12	Non-native	Drosophila_immigrans	19.568	-155.230
ITS-322_S64_L001	PRJNA1270093	UHM (this study)	United States	Hawai'i	H_Army_Rd	2020	12	Native	Drosophila_sproati	19.568	-155.230
ITS-323_S65_L001	PRJNA1270093	UHM (this study)	United States	Hawai'i	H_Army_Rd	2020	12	Native	Drosophila_sproati	19.568	-155.230
ITS-324_S66_L001	PRJNA1270093	UHM (this study)	United States	Hawai'i	H_Army_Rd	2020	12	Native	Drosophila_basisetae	19.568	-155.230
ITS-33_S119_L001	PRJNA1270093	UHM (this study)	United States	Hawai'i	H_HETF	2019	5	Native	Drosophila_sproati	19.912	-155.313
ITS-330_S72_L001	PRJNA1270093	UHM (this study)	United States	Hawai'i	H_Saddle_Rd	2020	12	Non-native	Drosophila_immigrans	19.675	-155.332
ITS-332_S74_L001	PRJNA1270093	UHM (this study)	United States	Hawai'i	H_Saddle_Rd	2020	12	Native	Drosophila_sproati	19.675	-155.332
ITS-335_S77_L001	PRJNA1270093	UHM (this study)	United States	Hawai'i	H_Saddle_Rd	2020	10	Native	Drosophila_medialis	19.675	-155.332
ITS-34_S120_L001	PRJNA1270093	UHM (this study)	United States	Hawai'i	H_HETF	2019	5	Native	Drosophila_sproati	19.912	-155.313
ITS-35_S121_L001	PRJNA1270093	UHM (this study)	United States	Hawai'i	H_HETF	2019	5	Native	Drosophila_murphyi	19.912	-155.313
ITS-36_S122_L001	PRJNA1270093	UHM (this study)	United States	Hawai'i	H_HETF	2019	5	Native	Drosophila_sproati	19.912	-155.313
ITS-37_S123_L001	PRJNA1270093	UHM (this study)	United States	Hawai'i	H_HETF	2019	5	Native	Drosophila_sproati	19.912	-155.313
ITS-38_S124_L001	PRJNA1270093	UHM (this study)	United States	Hawai'i	H_Toms_Trail	2020	10	Native	Drosophila_ochracea	19.574	-155.216
ITS-380_S234_L001	PRJNA1270093	UHM (this study)	United States	Hawai'i	H_KauFR	2021	3	Native	Drosophila_sproati	19.341	-155.460
ITS-381_S235_L001	PRJNA1270093	UHM (this study)	United States	Hawai'i	H_KauFR	2021	3	Native	Drosophila_sproati	19.341	-155.460
ITS-382_S236_L001	PRJNA1270093	UHM (this study)	United States	Hawai'i	H_KauFR	2021	3	Native	Drosophila_sproati	19.341	-155.460
ITS-383_S237_L001	PRJNA1270093	UHM (this study)	United States	Hawai'i	H_KauFR	2021	3	Native	Drosophila_murphyi	19.341	-155.460
ITS-384_S238_L001	PRJNA1270093	UHM (this study)	United States	Hawai'i	H_KauFR	2021	3	Native	Drosophila_murphyi	19.341	-155.460
ITS-385_S239_L001	PRJNA1270093	UHM (this study)	United States	Hawai'i	H_KauFR	2021	3	Native	Drosophila_murphyi	19.341	-155.460
ITS-393_S248_L001	PRJNA1270093	UHM (this study)	United States	Hawai'i	H_KauFR	2021	3	Non-native	Drosophila_immigrans	19.341	-155.460
ITS-394_S249_L001	PRJNA1270093	UHM (this study)	United States	Hawai'i	H_KauFR	2021	3	Non-native	Drosophila_immigrans	19.341	-155.460
ITS-395_S250_L001	PRJNA1270093	UHM (this study)	United States	Hawai'i	H_KauFR	2021	3	Non-native	Drosophila_immigrans	19.341	-155.460
ITS-396_S251_L001	PRJNA1270093	UHM (this study)	United States	Hawai'i	H_KauFR	2021	3	Non-native	Drosophila_immigrans	19.341	-155.460
ITS-399_S254_L001	PRJNA1270093	UHM (this study)	United States	Hawai'i	H_KauFR	2021	3	Native	Drosophila_murphyi	19.341	-155.460
ITS-40_S125_L001	PRJNA1270093	UHM (this study)	United States	Hawai'i	H_Toms_Trail	2020	12	Native	Drosophila_sproati	19.574	-155.216
ITS-400_S255_L001	PRJNA1270093	UHM (this study)	United States	Hawai'i	H_KauFR	2021	3	Native	Drosophila_murphyi	19.341	-155.460
ITS-401_S256_L001	PRJNA1270093	UHM (this study)	United States	Hawai'i	H_KauFR	2021	3	Native	Drosophila_murphyi	19.341	-155.460

Sample	Project	Source	Country	Locality_1	Locality_2[†]	Year	Month	Status	Species identification	Latitude	Longitude
ITS-402_S257_L001	PRJNA1270093	UHM (this study)	United States	Hawai'i	H_KauFR	2021	3	Native	Drosophila_murphyi	19.341	-155.460
ITS-403_S258_L001	PRJNA1270093	UHM (this study)	United States	Hawai'i	H_KauFR	2021	3	Native	Drosophila_murphyi	19.341	-155.460
ITS-404_S259_L001	PRJNA1270093	UHM (this study)	United States	Hawai'i	H_KauFR	2021	3	Native	Drosophila_sproati	19.341	-155.460
ITS-405_S260_L001	PRJNA1270093	UHM (this study)	United States	Hawai'i	H_KauFR	2021	3	Native	Drosophila_sproati	19.341	-155.460
ITS-406_S261_L001	PRJNA1270093	UHM (this study)	United States	Hawai'i	H_KauFR	2021	3	Native	Drosophila_sproati	19.341	-155.460
ITS-407_S262_L001	PRJNA1270093	UHM (this study)	United States	Hawai'i	H_KauFR	2021	3	Native	Drosophila_sproati	19.341	-155.460
ITS-408_S263_L001	PRJNA1270093	UHM (this study)	United States	Hawai'i	H_KauFR	2021	3	Non-native	Drosophila_immigrans	19.341	-155.460
ITS-412_S267_L001	PRJNA1270093	UHM (this study)	United States	Hawai'i	H_KauFR	2021	3	Non-native	Drosophila_immigrans	19.341	-155.460
ITS-500_S289_L001	PRJNA1270093	UHM (this study)	United States	Hawai'i	Mo_PuuKolekole	2021	5	Native	Drosophila_fasciculisetae	21.118	-156.908
ITS-501_S290_L001	PRJNA1270093	UHM (this study)	United States	Hawai'i	Mo_PuuKolekole	2021	5	Native	Drosophila_fasciculisetae	21.118	-156.908
ITS-502_S291_L001	PRJNA1270093	UHM (this study)	United States	Hawai'i	Mo_PuuKolekole	2021	5	Native	Drosophila_fasciculisetae	21.118	-156.908
ITS-503_S292_L001	PRJNA1270093	UHM (this study)	United States	Hawai'i	Mo_PuuKolekole	2021	5	Native	Drosophila_fasciculisetae	21.118	-156.908
ITS-504_S293_L001	PRJNA1270093	UHM (this study)	United States	Hawai'i	Mo_PuuKolekole	2021	5	Native	Drosophila_fasciculisetae	21.118	-156.908
ITS-505_S294_L001	PRJNA1270093	UHM (this study)	United States	Hawai'i	Mo_PuuKolekole	2021	5	Native	Drosophila_fasciculisetae	21.118	-156.908
ITS-506_S295_L001	PRJNA1270093	UHM (this study)	United States	Hawai'i	Mo_PuuKolekole	2021	5	Native	Drosophila_fasciculisetae	21.118	-156.908
ITS-507_S296_L001	PRJNA1270093	UHM (this study)	United States	Hawai'i	Mo_PuuKolekole	2021	5	Native	Drosophila_fasciculisetae	21.118	-156.908
ITS-508_S297_L001	PRJNA1270093	UHM (this study)	United States	Hawai'i	Mo_PuuKolekole	2021	5	Native	Drosophila_fasciculisetae	21.118	-156.908
ITS-509_S298_L001	PRJNA1270093	UHM (this study)	United States	Hawai'i	Mo_PuuKolekole	2021	5	Native	Drosophila_fasciculisetae	21.118	-156.908
ITS-510_S299_L001	PRJNA1270093	UHM (this study)	United States	Hawai'i	Mo_PuuKolekole	2021	5	Native	Drosophila_fasciculisetae	21.118	-156.908
ITS-511_S300_L001	PRJNA1270093	UHM (this study)	United States	Hawai'i	Mo_PuuKolekole	2021	5	Native	Drosophila_fasciculisetae	21.118	-156.908
ITS-512_S301_L001	PRJNA1270093	UHM (this study)	United States	Hawai'i	Mo_PuuKolekole	2021	5	Native	Drosophila_fasciculisetae	21.118	-156.908
ITS-513_S302_L001	PRJNA1270093	UHM (this study)	United States	Hawai'i	Mo_PuuKolekole	2021	5	Native	Drosophila_neoperkinsi	21.118	-156.908
ITS-514_S303_L001	PRJNA1270093	UHM (this study)	United States	Hawai'i	Mo_PuuKolekole	2021	5	Native	Drosophila_neoperkinsi	21.118	-156.908
ITS-515_S304_L001	PRJNA1270093	UHM (this study)	United States	Hawai'i	Mo_PuuKolekole	2021	5	Native	Drosophila_neoperkinsi	21.118	-156.908
ITS-516_S305_L001	PRJNA1270093	UHM (this study)	United States	Hawai'i	Mo_PuuKolekole	2021	5	Native	Drosophila_neoperkinsi	21.118	-156.908
ITS-517_S306_L001	PRJNA1270093	UHM (this study)	United States	Hawai'i	Mo_PuuKolekole	2021	5	Native	Drosophila_grimshawi	21.118	-156.908
ITS-522_S311_L001	PRJNA1270093	UHM (this study)	United States	Hawai'i	Mo_PuuKolekole	2021	5	Non-native	Drosophila_immigrans	21.118	-156.908
ITS-523_S312_L001	PRJNA1270093	UHM (this study)	United States	Hawai'i	Mo_PuuKolekole	2021	5	Non-native	Drosophila_suzukii	21.118	-156.908
ITS-524_S313_L001	PRJNA1270093	UHM (this study)	United States	Hawai'i	Mo_PuuKolekole	2021	5	Non-native	Drosophila_immigrans	21.118	-156.908
ITS-530_S319_L001	PRJNA1270093	UHM (this study)	United States	Hawai'i	Mo_Pepeopae	2021	5	Native	Drosophila_fasciculisetae	21.118	-156.909
ITS-531_S320_L001	PRJNA1270093	UHM (this study)	United States	Hawai'i	Mo_Pepeopae	2021	5	Native	Drosophila_fasciculisetae	21.118	-156.909
ITS-534_S323_L001	PRJNA1270093	UHM (this study)	United States	Hawai'i	Mo_Hanalilolilo	2021	5	Native	Drosophila_bostrycha	21.126	-156.915
ITS-535_S324_L001	PRJNA1270093	UHM (this study)	United States	Hawai'i	Mo_Hanalilolilo	2021	5	Native	Drosophila_neoperkinsi	21.126	-156.915
ITS-536_S325_L001	PRJNA1270093	UHM (this study)	United States	Hawai'i	Mo_Hanalilolilo	2021	5	Native	Drosophila_neoperkinsi	21.126	-156.915
ITS-537_S326_L001	PRJNA1270093	UHM (this study)	United States	Hawai'i	Mo_Hanalilolilo	2021	5	Native	Drosophila_bostrycha	21.126	-156.915
ITS-538_S327_L001	PRJNA1270093	UHM (this study)	United States	Hawai'i	Mo_Hanalilolilo	2021	5	Native	Drosophila_cilifera	21.126	-156.915
ITS-539_S328_L001	PRJNA1270093	UHM (this study)	United States	Hawai'i	Mo_Hanalilolilo	2021	5	Native	Drosophila_fasciculisetae	21.126	-156.915
ITS-540_S329_L001	PRJNA1270093	UHM (this study)	United States	Hawai'i	Mo_Hanalilolilo	2021	5	Native	Drosophila_fasciculisetae	21.126	-156.915
ITS-541_S330_L001	PRJNA1270093	UHM (this study)	United States	Hawai'i	Mo_Hanalilolilo	2021	5	Native	Drosophila_fasciculisetae	21.126	-156.915
ITS-542_S331_L001	PRJNA1270093	UHM (this study)	United States	Hawai'i	Mo_Hanalilolilo	2021	5	Native	Drosophila_fasciculisetae	21.126	-156.915
ITS-543_S332_L001	PRJNA1270093	UHM (this study)	United States	Hawai'i	Mo_Hanalilolilo	2021	5	Native	Drosophila_fasciculisetae	21.126	-156.915
ITS-545_S334_L001	PRJNA1270093	UHM (this study)	United States	Hawai'i	Mo_Hanalilolilo	2021	5	Non-native	Drosophila_immigrans	21.126	-156.915
ITS-546_S335_L001	PRJNA1270093	UHM (this study)	United States	Hawai'i	Mo_Hanalilolilo	2021	5	Non-native	Drosophila_immigrans	21.126	-156.915
ITS-558_S347_L001	PRJNA1270093	UHM (this study)	United States	Hawai'i	Mo_Hanalilolilo	2021	5	Non-native	Drosophila_suzukii	21.126	-156.915
ITS-567_S356_L001	PRJNA1270093	UHM (this study)	United States	Hawai'i	H_Olaa_Pole44	2021	3	Native	Drosophila_sproati	19.462	-155.248
ITS-568_S357_L001	PRJNA1270093	UHM (this study)	United States	Hawai'i	H_Olaa_Pole44	2021	3	Native	Drosophila_sproati	19.462	-155.248
ITS-569_S358_L001	PRJNA1270093	UHM (this study)	United States	Hawai'i	H_Olaa_Pole44	2021	3	Native	Drosophila_sproati	19.462	-155.248
ITS-570_S359_L001	PRJNA1270093	UHM (this study)	United States	Hawai'i	H_Olaa_Pole44	2021	3	Native	Drosophila_sproati	19.462	-155.248
ITS-571_S360_L001	PRJNA1270093	UHM (this study)	United States	Hawai'i	H_Olaa_Pole44	2021	3	Native	Drosophila_setosimentum	19.462	-155.248
ITS-581_S370_L001	PRJNA1270093	UHM (this study)	United States	Hawai'i	H_Olaa_Pole44	2021	3	Non-native	Drosophila_immigrans	19.462	-155.248
ITS-583_S372_L001	PRJNA1270093	UHM (this study)	United States	Hawai'i	H_Olaa_Pole44	2021	3	Non-native	Drosophila_immigrans	19.462	-155.248
ITS-584_S373_L001	PRJNA1270093	UHM (this study)	United States	Hawai'i	H_Olaa_Pole44	2021	3	Non-native	Drosophila_immigrans	19.462	-155.248
ITS-592_S381_L001	PRJNA1270093	UHM (this study)	United States	Hawai'i	H_Olaa_Pole44	2021	3	Non-native	Drosophila_immigrans	19.462	-155.248
ITS-596_S388_L001	PRJNA1270093	UHM (this study)	United States	Hawai'i	H_Olaa_Pole44	2021	3	Native	Drosophila_sproati	19.462	-155.248
ITS-597_S389_L001	PRJNA1270093	UHM (this study)	United States	Hawai'i	H_Olaa_Pole44	2021	3	Native	Drosophila_sproati	19.462	-155.248
ITS-598_S390_L001	PRJNA1270093	UHM (this study)	United States	Hawai'i	H_Olaa_Pole44	2021	3	Native	Drosophila_sproati	19.462	-155.248
ITS-599_S391_L001	PRJNA1270093	UHM (this study)	United States	Hawai'i	H_Olaa_Pole44	2021	3	Native	Drosophila_sproati	19.462	-155.248
ITS-600_S392_L001	PRJNA1270093	UHM (this study)	United States	Hawai'i	H_Olaa_Pole44	2021	3	Native	Drosophila_prolaticilia	19.462	-155.248
ITS-601_S393_L001	PRJNA1270093	UHM (this study)	United States	Hawai'i	H_Olaa_Pole44	2021	3	Native	Drosophila_basisetae	19.462	-155.248
ITS-602_S394_L001	PRJNA1270093	UHM (this study)	United States	Hawai'i	H_Olaa_Pole44	2021	3	Native	Drosophila_basisetae	19.462	-155.248
ITS-603_S395_L001	PRJNA1270093	UHM (this study)	United States	Hawai'i	H_Olaa_Pole44	2021	3	Native	Drosophila_basisetae	19.462	-155.248
ITS-604_S396_L001	PRJNA1270093	UHM (this study)	United States	Hawai'i	H_Olaa_Pole44	2021	3	Native	Drosophila_basisetae	19.462	-155.248

Sample	Project	Source	Country	Locality_1	Locality_2[†]	Year	Month	Status	Species identification	Latitude	Longitude
ITS-606_S398_L001	PRJNA1270093	UHM (this study)	United States	Hawai'i	H_Puu_Makaala	2021	3	Non-native	Drosophila_immigrans	19.484	-155.271
ITS-607_S399_L001	PRJNA1270093	UHM (this study)	United States	Hawai'i	H_Puu_Makaala	2021	3	Non-native	Drosophila_immigrans	19.484	-155.271
ITS-608_S400_L001	PRJNA1270093	UHM (this study)	United States	Hawai'i	H_Puu_Makaala	2021	3	Non-native	Drosophila_immigrans	19.484	-155.271
ITS-610_S402_L001	PRJNA1270093	UHM (this study)	United States	Hawai'i	H_Puu_Makaala	2021	3	Non-native	Drosophila_immigrans	19.484	-155.271
ITS-611_S403_L001	PRJNA1270093	UHM (this study)	United States	Hawai'i	H_Puu_Makaala	2021	3	Native	Drosophila_sproati	19.484	-155.271
ITS-612_S404_L001	PRJNA1270093	UHM (this study)	United States	Hawai'i	H_Puu_Makaala	2021	3	Native	Drosophila_sproati	19.484	-155.271
ITS-613_S405_L001	PRJNA1270093	UHM (this study)	United States	Hawai'i	H_Puu_Makaala	2021	3	Native	Drosophila_sproati	19.484	-155.271
ITS-614_S406_L001	PRJNA1270093	UHM (this study)	United States	Hawai'i	H_Puu_Makaala	2021	3	Native	Drosophila_sproati	19.484	-155.271
ITS-615_S407_L001	PRJNA1270093	UHM (this study)	United States	Hawai'i	H_Puu_Makaala	2021	3	Native	Drosophila_sproati	19.484	-155.271
ITS-616_S408_L001	PRJNA1270093	UHM (this study)	United States	Hawai'i	H_Puu_Makaala	2021	3	Native	Drosophila_sproati	19.484	-155.271
ITS-617_S409_L001	PRJNA1270093	UHM (this study)	United States	Hawai'i	H_Puu_Makaala	2021	3	Native	Drosophila_sproati	19.484	-155.271
ITS-619_S411_L001	PRJNA1270093	UHM (this study)	United States	Hawai'i	H_Puu_Makaala	2021	3	Native	Drosophila_setosimentum	19.484	-155.271
ITS-620_S412_L001	PRJNA1270093	UHM (this study)	United States	Hawai'i	H_Puu_Makaala	2021	3	Native	Drosophila_setosimentum	19.484	-155.271
ITS-621_S413_L001	PRJNA1270093	UHM (this study)	United States	Hawai'i	H_Puu_Makaala	2021	3	Native	Drosophila_setosimentum	19.484	-155.271
ITS-629_S421_L001	PRJNA1270093	UHM (this study)	United States	Hawai'i	H_Kipuka_Ki	2021	3	Non-native	Drosophila_suzukii	19.442	-155.316
ITS-631_S423_L001	PRJNA1270093	UHM (this study)	United States	Hawai'i	H_Kipuka_Ki	2021	3	Non-native	Drosophila_suzukii	19.442	-155.316
ITS-632_S424_L001	PRJNA1270093	UHM (this study)	United States	Hawai'i	H_Kipuka_Ki	2021	3	Non-native	Drosophila_suzukii	19.442	-155.316
ITS-643_S435_L001	PRJNA1270093	UHM (this study)	United States	Hawai'i	H_Olaa_Pole44	2021	3	Native	Drosophila_ochracea	19.462	-155.248
ITS-644_S436_L001	PRJNA1270093	UHM (this study)	United States	Hawai'i	H_Olaa_Pole44	2021	3	Native	Drosophila_ochracea	19.462	-155.248
ITS-645_S437_L001	PRJNA1270093	UHM (this study)	United States	Hawai'i	L_Puhielelu_exclosure	2021	7	Native	Drosophila_grimshawi	20.806	-156.862
ITS-646_S438_L001	PRJNA1270093	UHM (this study)	United States	Hawai'i	L_Puhielelu_exclosure	2021	7	Non-native	Drosophila_immigrans	20.806	-156.862
ITS-647_S439_L001	PRJNA1270093	UHM (this study)	United States	Hawai'i	L_Puhielelu_exclosure	2021	7	Non-native	Drosophila_immigrans	20.806	-156.862
ITS-648_S440_L001	PRJNA1270093	UHM (this study)	United States	Hawai'i	L_Puhielelu_exclosure	2021	7	Non-native	Drosophila_immigrans	20.806	-156.862
ITS-649_S441_L001	PRJNA1270093	UHM (this study)	United States	Hawai'i	L_Puhielelu_exclosure	2021	7	Non-native	Drosophila_immigrans	20.806	-156.862
ITS-651_S443_L001	PRJNA1270093	UHM (this study)	United States	Hawai'i	L_Puhielelu_parking	2021	7	Non-native	Drosophila_immigrans	20.806	-156.865
ITS-652_S444_L001	PRJNA1270093	UHM (this study)	United States	Hawai'i	L_Puhielelu_parking	2021	7	Non-native	Drosophila_suzukii	20.806	-156.865
ITS-653_S445_L001	PRJNA1270093	UHM (this study)	United States	Hawai'i	L_Lanaihale	2021	7	Non-native	Drosophila_immigrans	20.813	-156.874
ITS-654_S446_L001	PRJNA1270093	UHM (this study)	United States	Hawai'i	L_Lanaihale	2021	7	Non-native	Drosophila_immigrans	20.813	-156.874
ITS-656_S448_L001	PRJNA1270093	UHM (this study)	United States	Hawai'i	L_Lanaihale	2021	7	Non-native	Drosophila_immigrans	20.813	-156.874
ITS-657_S449_L001	PRJNA1270093	UHM (this study)	United States	Hawai'i	L_Lanaihale	2021	7	Non-native	Drosophila_suzukii	20.813	-156.874
ITS-658_S450_L001	PRJNA1270093	UHM (this study)	United States	Hawai'i	L_Lanaihale	2021	7	Non-native	Drosophila_suzukii	20.813	-156.874
ITS-659_S451_L001	PRJNA1270093	UHM (this study)	United States	Hawai'i	L_Lanaihale	2021	7	Non-native	Drosophila_suzukii	20.813	-156.874
ITS-71_S145_L001	PRJNA1270093	UHM (this study)	United States	Hawai'i	H_Toms_Trail	2020	10	Native	Drosophila_ochracea	19.574	-155.216
ITS-74_S146_L001	PRJNA1270093	UHM (this study)	United States	Hawai'i	H_Toms_Trail	2020	12	Native	Drosophila_basisetae	19.574	-155.216
ITS-77_S147_L001	PRJNA1270093	UHM (this study)	United States	Hawai'i	H_Toms_Trail	2020	10	Native	Drosophila_ochracea	19.574	-155.216
ITS-78_S148_L001	PRJNA1270093	UHM (this study)	United States	Hawai'i	H_Toms_Trail	2020	10	Native	Drosophila_ochracea	19.574	-155.216
ITS-8_S102_L001	PRJNA1270093	UHM (this study)	United States	Hawai'i	H_Toms_Trail	2020	10	Native	Drosophila_ochracea	19.574	-155.216
ITS-80_S149_L001	PRJNA1270093	UHM (this study)	United States	Hawai'i	H_Toms_Trail	2020	10	Native	Drosophila_ochracea	19.574	-155.216
ITS-81_S150_L001	PRJNA1270093	UHM (this study)	United States	Hawai'i	H_Toms_Trail	2020	10	Native	Drosophila_ochracea	19.574	-155.216
ITS-85_S151_L001	PRJNA1270093	UHM (this study)	United States	Hawai'i	H_Toms_Trail	2020	10	Native	Drosophila_sproati	19.574	-155.216
ITS-88_S152_L001	PRJNA1270093	UHM (this study)	United States	Hawai'i	H_Toms_Trail	2020	12	Native	Drosophila_basisetae	19.574	-155.216
ITS-89_S153_L001	PRJNA1270093	UHM (this study)	United States	Hawai'i	H_Toms_Trail	2020	12	Native	Drosophila_basisetae	19.574	-155.216
ITS-9_S103_L001	PRJNA1270093	UHM (this study)	United States	Hawai'i	H_Toms_Trail	2020	10	Native	Drosophila_ochracea	19.574	-155.216
ITS-93_S154_L001	PRJNA1270093	UHM (this study)	United States	Hawai'i	H_Toms_Trail	2020	12	Native	Drosophila_basisetae	19.574	-155.216

[†] Mo: Molokai; H: Hawaii Island; L: Lanai

Table S3. Summary of multi-region *D. suzukii* data sets, collection sites, and samples used for 16S rRNA analysis.

Name	Project	Source[1]	Country	Locality_1	Locality_2[†]	Locality_3	Year	Month†	Status	Code
SRR14172012	PRJNA719706	Lin	China	China	Taian_China	China+Japan	2016	5	lab	NA
SRR14172015	PRJNA719706	Lin	China	China	Taian_China	China+Japan	2016	5	lab	NA
SRR14172016	PRJNA719706	Lin	China	China	Taian_China	China+Japan	2016	5	lab	NA
SRR4417507	PRJNA347319	Martinez-Sanudo	Japan	Japan	Tokyo	China+Japan	2015	6	native	NA
SRR4417508	PRJNA347319	Martinez-Sanudo	Japan	Japan	Tokyo	China+Japan	2015	6	native	NA
SRR4417509	PRJNA347319	Martinez-Sanudo	Italy	Italy	Verona	Europe	2014	8	non-native	NA
SRR4417510	PRJNA347319	Martinez-Sanudo	Italy	Italy	Verona	Europe	2014	8	non-native	NA
SRR4417511	PRJNA347319	Martinez-Sanudo	United States	United States	Oregon	US	2014	8	non-native	NA
SRR4417512	PRJNA347319	Martinez-Sanudo	United States	United States	Oregon	US	2014	8	non-native	NA
SRR4417513	PRJNA347319	Martinez-Sanudo	France	France	Cap Esterel	Europe	2014	9	non-native	NA
SRR4417514	PRJNA347319	Martinez-Sanudo	France	France	Cap Esterel	Europe	2014	9	non-native	NA
SRR4417515	PRJNA347319	Martinez-Sanudo	Slovenia	Slovenia	Mojstrana	Europe	2014	10	non-native	NA
SRR4417516	PRJNA347319	Martinez-Sanudo	Slovenia	Slovenia	Mojstrana	Europe	2014	10	non-native	NA
SRR4417517	PRJNA347319	Martinez-Sanudo	Switzerland	Switzerland	Delemont	Europe	2014	8	non-native	NA
SRR4417518	PRJNA347319	Martinez-Sanudo	Switzerland	Switzerland	Delemont	Europe	2014	8	non-native	NA
SRR4417519	PRJNA347319	Martinez-Sanudo	China	China	Miaofengshan-Beijing	China+Japan	2015	7	native	NA
SRR4417520	PRJNA347319	Martinez-Sanudo	United Kingdom	United Kingdom	York	UK	2014	11	non-native	NA
SRR4417521	PRJNA347319	Martinez-Sanudo	United Kingdom	United Kingdom	York	UK	2014	11	non-native	NA
SRR4417522	PRJNA347319	Martinez-Sanudo	Italy	Italy	Pergine_Italy	Europe	2014	6	lab	NA
SRR4417523	PRJNA347319	Martinez-Sanudo	China	China	Miaofengshan-Beijing	China+Japan	2015	7	native	NA
SRR4417524	PRJNA347319	Martinez-Sanudo	China	China	Liaoyuan-Jilin	China+Japan	2014	7	native	NA
SRR4417525	PRJNA347319	Martinez-Sanudo	China	China	Liaoyuan-Jilin	China+Japan	2014	7	native	NA
SRR4417526	PRJNA347319	Martinez-Sanudo	China	China	Kunming- Yunnan	China+Japan	2015	6	native	NA
SRR4417527	PRJNA347319	Martinez-Sanudo	China	China	Kunming- Yunnan	China+Japan	2015	6	native	NA
SRR4417528	PRJNA347319	Martinez-Sanudo	Spain	Spain	Valencia	Europe	2014	9	non-native	NA
SRR4417529	PRJNA347319	Martinez-Sanudo	Spain	Spain	Valencia	Europe	2014	9	non-native	NA
16S_523_S24_L001	PRJNA1270093	UHM (this study)	United States	Hawaii	Mo_PuuKolekole	Hawaii	2021	5	non-native	NA
16S_558_S59_L001	PRJNA1270093	UHM (this study)	United States	Hawaii	Mo_Hanalilolilo	Hawaii	2021	5	non-native	NA
16S_629_S133_L001	PRJNA1270093	UHM (this study)	United States	Hawaii	H_Kipuka_Ki	Hawaii	2021	6	non-native	NA
16S_631_S135_L001	PRJNA1270093	UHM (this study)	United States	Hawaii	H_Kipuka_Ki	Hawaii	2021	6	non-native	NA
16S_632_S136_L001	PRJNA1270093	UHM (this study)	United States	Hawaii	H_Kipuka_Ki	Hawaii	2021	6	non-native	NA
16S_652_S156_L001	PRJNA1270093	UHM (this study)	United States	Hawaii	L_Puhielelu_parking	Hawaii	2021	6	non-native	NA
16S_657_S161_L001	PRJNA1270093	UHM (this study)	United States	Hawaii	L_Lanaihale	Hawaii	2021	6	non-native	NA
16S_658_S162_L001	PRJNA1270093	UHM (this study)	United States	Hawaii	L_Lanaihale	Hawaii	2021	6	non-native	NA
16S_659_S163_L001	PRJNA1270093	UHM (this study)	United States	Hawaii	L_Lanaihale	Hawaii	2021	6	non-native	NA
ERR8054585	PRJEB50289	Fountain	United Kingdom	United Kingdom	NA	UK	2015	NA	non-native	NA
ERR8054586	PRJEB50289	Fountain	United Kingdom	United Kingdom	NA	UK	2015	NA	non-native	NA
ERR8054588	PRJEB50289	Fountain	United Kingdom	United Kingdom	NA	UK	2015	NA	non-native	NA
ERR8054589	PRJEB50289	Fountain	United Kingdom	United Kingdom	NA	UK	2015	NA	non-native	NA
ERR8054590	PRJEB50289	Fountain	United Kingdom	United Kingdom	NA	UK	2015	NA	non-native	NA
ERR8054591	PRJEB50289	Fountain	United Kingdom	United Kingdom	NA	UK	2015	NA	non-native	NA
ERR8054592	PRJEB50289	Fountain	United Kingdom	United Kingdom	NA	UK	2015	NA	non-native	NA
ERR8054593	PRJEB50289	Fountain	United Kingdom	United Kingdom	NA	UK	2015	NA	non-native	NA
ERR8054594	PRJEB50289	Fountain	United Kingdom	United Kingdom	NA	UK	2015	NA	non-native	NA

NAME	Project	Source[1]	Country	Locality_1	Locality_2	Locality_3	Year	Month	Status	Code
ERR8054595	PRJEB50289	Fountain	United Kingdom	United Kingdom	NA	UK	2015	NA	non-native	NA
ERR8054596	PRJEB50289	Fountain	United Kingdom	United Kingdom	NA	UK	2015	NA	non-native	NA
ERR8054597	PRJEB50289	Fountain	United Kingdom	United Kingdom	NA	UK	2015	NA	non-native	NA
ERR8054598	PRJEB50289	Fountain	United Kingdom	United Kingdom	NA	UK	2015	NA	non-native	NA
ERR8054599	PRJEB50289	Fountain	United Kingdom	United Kingdom	NA	UK	2015	NA	non-native	NA
ERR8054601	PRJEB50289	Fountain	United Kingdom	United Kingdom	NA	UK	2015	NA	non-native	NA
ERR8054602	PRJEB50289	Fountain	United Kingdom	United Kingdom	NA	UK	2015	NA	non-native	NA
ERR8054603	PRJEB50289	Fountain	United Kingdom	United Kingdom	NA	UK	2015	NA	non-native	NA
ERR8054606	PRJEB50289	Fountain	United Kingdom	United Kingdom	NA	UK	2015	NA	non-native	NA
ERR8054607	PRJEB50289	Fountain	United Kingdom	United Kingdom	NA	UK	2015	NA	non-native	NA
ERR8054608	PRJEB50289	Fountain	United Kingdom	United Kingdom	NA	UK	2015	NA	non-native	NA
ERR8054609	PRJEB50289	Fountain	United Kingdom	United Kingdom	NA	UK	2015	NA	non-native	NA
ERR8054610	PRJEB50289	Fountain	United Kingdom	United Kingdom	NA	UK	2015	NA	non-native	NA
ERR8054612	PRJEB50289	Fountain	United Kingdom	United Kingdom	NA	UK	2015	NA	non-native	NA
ERR8054613	PRJEB50289	Fountain	United Kingdom	United Kingdom	NA	UK	2015	NA	non-native	NA
ERR8054614	PRJEB50289	Fountain	United Kingdom	United Kingdom	NA	UK	2015	NA	non-native	NA
ERR8054615	PRJEB50289	Fountain	United Kingdom	United Kingdom	NA	UK	2016	NA	non-native	NA
ERR8054617	PRJEB50289	Fountain	United Kingdom	United Kingdom	NA	UK	2016	NA	non-native	NA
ERR8054619	PRJEB50289	Fountain	United Kingdom	United Kingdom	NA	UK	2016	NA	non-native	NA
ERR8054620	PRJEB50289	Fountain	United Kingdom	United Kingdom	NA	UK	2016	NA	non-native	NA
ERR8054717	PRJEB50289	Fountain	United Kingdom	United Kingdom	NA	UK	2016	NA	non-native	NA
ERR8054719	PRJEB50289	Fountain	United Kingdom	United Kingdom	NA	UK	2016	NA	non-native	NA
ERR8054720	PRJEB50289	Fountain	United Kingdom	United Kingdom	NA	UK	2016	NA	non-native	NA
ERR8054721	PRJEB50289	Fountain	United Kingdom	United Kingdom	NA	UK	2016	NA	non-native	NA
ERR8054722	PRJEB50289	Fountain	United Kingdom	United Kingdom	NA	UK	2016	NA	non-native	NA
ERR8054724	PRJEB50289	Fountain	United Kingdom	United Kingdom	NA	UK	2016	NA	non-native	NA
ERR8054725	PRJEB50289	Fountain	United Kingdom	United Kingdom	NA	UK	2016	NA	non-native	NA
ERR8054726	PRJEB50289	Fountain	United Kingdom	United Kingdom	NA	UK	2016	NA	non-native	NA
ERR8054727	PRJEB50289	Fountain	United Kingdom	United Kingdom	NA	UK	2016	NA	non-native	NA
ERR8054729	PRJEB50289	Fountain	United Kingdom	United Kingdom	NA	UK	2016	NA	non-native	NA
ERR8054730	PRJEB50289	Fountain	United Kingdom	United Kingdom	NA	UK	2016	NA	non-native	NA
ERR8054731	PRJEB50289	Fountain	United Kingdom	United Kingdom	NA	UK	2016	NA	non-native	NA
ERR8054732	PRJEB50289	Fountain	United Kingdom	United Kingdom	NA	UK	2016	NA	non-native	NA
ERR8054735	PRJEB50289	Fountain	United Kingdom	United Kingdom	NA	UK	2016	NA	non-native	NA
ERR8054736	PRJEB50289	Fountain	United Kingdom	United Kingdom	NA	UK	2016	NA	non-native	NA
ERR8054737	PRJEB50289	Fountain	United Kingdom	United Kingdom	NA	UK	2016	NA	non-native	NA
ERR8054738	PRJEB50289	Fountain	United Kingdom	United Kingdom	NA	UK	2016	NA	non-native	NA
ERR8054740	PRJEB50289	Fountain	United Kingdom	United Kingdom	NA	UK	2016	NA	non-native	NA
ERR8054742	PRJEB50289	Fountain	United Kingdom	United Kingdom	NA	UK	2016	NA	non-native	NA
ERR8054744	PRJEB50289	Fountain	United Kingdom	United Kingdom	NA	UK	2016	NA	non-native	NA
ERR8054745	PRJEB50289	Fountain	United Kingdom	United Kingdom	NA	UK	2016	NA	non-native	NA
ERR8054746	PRJEB50289	Fountain	United Kingdom	United Kingdom	NA	UK	2016	NA	non-native	NA
ERR8054747	PRJEB50289	Fountain	United Kingdom	United Kingdom	NA	UK	2016	NA	non-native	NA
ERR8054748	PRJEB50289	Fountain	United Kingdom	United Kingdom	NA	UK	2016	NA	non-native	NA
ERR8054749	PRJEB50289	Fountain	United Kingdom	United Kingdom	NA	UK	2016	NA	non-native	NA
ERR8054750	PRJEB50289	Fountain	United Kingdom	United Kingdom	NA	UK	2016	NA	non-native	NA

NAME	Project	Source[1]	Country	Locality_1	Locality_2	Locality_3	Year	Month	Status	Code
ERR8054751	PRJEB50289	Fountain	United Kingdom	United Kingdom	NA	UK	2016	NA	non-native	NA
ERR8054752	PRJEB50289	Fountain	United Kingdom	United Kingdom	NA	UK	2016	NA	non-native	NA
ERR8054753	PRJEB50289	Fountain	United Kingdom	United Kingdom	NA	UK	2016	NA	non-native	NA
ERR8054754	PRJEB50289	Fountain	United Kingdom	United Kingdom	NA	UK	2016	NA	non-native	NA
ERR8054756	PRJEB50289	Fountain	United Kingdom	United Kingdom	NA	UK	2016	NA	non-native	NA
ERR8054971	PRJEB50289	Fountain	United Kingdom	United Kingdom	NA	UK	2016	NA	non-native	NA
ERR8054973	PRJEB50289	Fountain	United Kingdom	United Kingdom	NA	UK	2016	NA	non-native	NA
ERR8054974	PRJEB50289	Fountain	United Kingdom	United Kingdom	NA	UK	2016	NA	non-native	NA
SRR6130729	PRJNA412893	Cornell Univ	United States	New York	New York	US_lab	2016	12	lab	NYB.F.2
SRR6130730	PRJNA412893	Cornell Univ	United States	New York	Geneva	US_wild	2016	9	non-native	lo.f.1.2
SRR6130731	PRJNA412893	Cornell Univ	United States	New York	New York	US_lab	2016	12	lab	NYB.F.1
SRR6130732	PRJNA412893	Cornell Univ	United States	New York	Geneva	US_wild	2016	9	non-native	lo.f.1.1
SRR6130733	PRJNA412893	Cornell Univ	United States	New York	Geneva	US_wild	2016	9	non-native	RPE.m.1.3
SRR6130734	PRJNA412893	Cornell Univ	United States	New York	Geneva	US_wild	2016	9	non-native	RPE.f.1.2
SRR6130735	PRJNA412893	Cornell Univ	United States	New York	Geneva	US_wild	2016	11	non-native	lo.m.2.1
SRR6130736	PRJNA412893	Cornell Univ	United States	New York	Geneva	US_wild	2016	9	non-native	lo.m.1.3
SRR6130737	PRJNA412893	Cornell Univ	United States	New York	Geneva	US_wild	2016	11	non-native	RPE.m.2.1
SRR6130738	PRJNA412893	Cornell Univ	United States	California	California	US_lab	2016	12	lab	CALI.F.3
SRR6130739	PRJNA412893	Cornell Univ	United States	New York	New York	US_lab	2016	12	lab	NYB.M.2
SRR6130740	PRJNA412893	Cornell Univ	United States	New York	Geneva	US_wild	2016	11	non-native	lo.f.2.3
SRR6130741	PRJNA412893	Cornell Univ	United States	New York	Geneva	US_wild	2016	9	non-native	lo.m.1.2
SRR6130742	PRJNA412893	Cornell Univ	United States	New York	Geneva	US_wild	2016	11	non-native	RPE.f.2.3
SRR6130743	PRJNA412893	Cornell Univ	United States	California	California	US_lab	2016	12	lab	CALI.M.1
SRR6130744	PRJNA412893	Cornell Univ	United States	New York	New York	US_lab	2016	12	lab	NYB.M.3
SRR6130745	PRJNA412893	Cornell Univ	United States	New York	Geneva	US_wild	2016	11	non-native	RPE.f.2.2
SRR6130746	PRJNA412893	Cornell Univ	United States	California	California	US_lab	2016	12	lab	CALI.F.1
SRR6130747	PRJNA412893	Cornell Univ	United States	New York	Geneva	US_wild	2016	11	non-native	lo.f.2.2
SRR6130748	PRJNA412893	Cornell Univ	United States	New York	Geneva	US_wild	2016	9	non-native	lo.m.1.1
SRR6130749	PRJNA412893	Cornell Univ	United States	New York	Geneva	US_wild	2016	9	non-native	lo.f.1.3
SRR6130750	PRJNA412893	Cornell Univ	United States	New York	Geneva	US_wild	2016	11	non-native	RPE.f.2.1
SRR6130751	PRJNA412893	Cornell Univ	United States	New York	New York	US_lab	2016	12	lab	NYB.F.3
SRR6130752	PRJNA412893	Cornell Univ	United States	New York	Geneva	US_wild	2016	11	non-native	lo.f.2.1
SRR6130753	PRJNA412893	Cornell Univ	United States	California	California	US_lab	2016	12	lab	CALI.F.2
SRR6130754	PRJNA412893	Cornell Univ	United States	New York	New York	US_lab	2016	12	lab	NYB.M.1
SRR6130755	PRJNA412893	Cornell Univ	United States	New York	Geneva	US_wild	2016	9	non-native	RPE.f.1.1
SRR6130756	PRJNA412893	Cornell Univ	United States	New York	Geneva	US_wild	2016	9	non-native	RPE.m.1.2
SRR6130757	PRJNA412893	Cornell Univ	United States	New York	Geneva	US_wild	2016	11	non-native	lo.m.2.3
SRR6130758	PRJNA412893	Cornell Univ	United States	California	California	US_lab	2016	12	lab	CALI.M.3
SRR6130759	PRJNA412893	Cornell Univ	United States	New York	Geneva	US_wild	2016	11	non-native	RPE.m.2.3
SRR6130760	PRJNA412893	Cornell Univ	United States	New York	Geneva	US_wild	2016	9	non-native	RPE.m.1.1
SRR6130761	PRJNA412893	Cornell Univ	United States	New York	Geneva	US_wild	2016	11	non-native	lo.m.2.2
SRR6130762	PRJNA412893	Cornell Univ	United States	California	California	US_lab	2016	12	lab	CALI.M.2
SRR6130763	PRJNA412893	Cornell Univ	United States	New York	Geneva	US_wild	2016	11	non-native	RPE.m.2.2
SRR6130764	PRJNA412893	Cornell Univ	United States	New York	Geneva	US_wild	2016	9	non-native	RPE.f.1.3

[1]Lin et al., 2021; Martinez-Sanudo et al., 2018; Fountain et al. 2018; UHM: University of Hawaiʻi at Mānoa

† Mo: Molokai; H: Hawaii Island; L: Lanai; NA: not available

Table S4. Pre-processing parameters for five independent sequencing projects used for bacterial taxonomic profiling of *Drosophila*.

	PRJEB50289	PRJNA347319	PRJNA412893	PRJNA719706	HI Dro[2]
**Primer set**	341F/ 805R	16S_univFor/ 16S_univRev	341F/ 785R	341F/ 805R	515F/ 806R
**Sequence type[1]**	paired-end	extended fragments	paired-end	extended fragments	paired-end
**trimLeft**	(18, 22)	19	(18, 25)	none	none
**truncLen**	(280, 220)	437	(275, 250)	none	(220, 190)
**maxEE**	(2, 2)	2	(2, 5)	2	(2, 2)

[1]For paired-end reads the trimming, truncation and maxEE parameters given in parentheses refer to the forward and reverse reads, respectively.
[2]*D. suzukii, D. immigrans,* and native Hawaiian *Drosophila* samples collected in Hawai'i.

Table S5. Read tracking through the taxonomy analysis pipeline for all samples in the five independent sources of sequencing data used in this analysis. Column headers are as follows: Input refers to raw sequence reads, Filtered refers to reads passing the pre-processing filters defined in Table S4, Raw ASVs refers to the reads remaining after dereplication and denoising in dada2, Tabled ASVs refers to the reads that were used to assign taxonomies following filtering in phyloseq, and Rarefied indicates that each sample was rarefied with a subsampling depth of 5,000 ASVs and showing which samples were dropped from the analysis (indicated by zero).

Project	Sample	Input	Filtered	Raw ASVs	Tabled ASVs	Rarefied
PRJEB50289	ERR8054585	60886	28354	21889	21032	5000
PRJEB50289	ERR8054586	69450	44028	42079	41360	5000
PRJEB50289	ERR8054588	25982	12606	12076	12041	5000
PRJEB50289	ERR8054589	76111	45393	40043	39757	5000
PRJEB50289	ERR8054590	50941	18311	12118	11605	5000
PRJEB50289	ERR8054591	27042	10890	6135	5706	5000
PRJEB50289	ERR8054592	29718	17482	13198	13118	5000
PRJEB50289	ERR8054593	12955	7292	6844	6577	5000
PRJEB50289	ERR8054594	62386	25903	18861	18118	5000
PRJEB50289	ERR8054595	174014	84738	61076	57991	5000
PRJEB50289	ERR8054596	392247	247450	238910	229364	5000
PRJEB50289	ERR8054597	1516	51	0	0	0
PRJEB50289	ERR8054598	1143	56	0	0	0
PRJEB50289	ERR8054599	105908	37688	5984	4569	5000
PRJEB50289	ERR8054601	54927	19968	15323	12627	5000
PRJEB50289	ERR8054602	123559	55678	32922	31979	5000
PRJEB50289	ERR8054603	187616	94046	63305	61708	5000
PRJEB50289	ERR8054606	120854	72246	59730	59471	5000
PRJEB50289	ERR8054607	75927	27934	5961	5552	5000
PRJEB50289	ERR8054608	154096	92910	81671	80024	5000
PRJEB50289	ERR8054609	128581	62533	45737	43186	5000
PRJEB50289	ERR8054610	51078	20767	9200	2690	5000
PRJEB50289	ERR8054612	47017	21808	13825	10049	5000
PRJEB50289	ERR8054613	89380	49602	36780	34583	5000
PRJEB50289	ERR8054614	55136	14701	128	128	0
PRJEB50289	ERR8054615	54938	37642	18078	17430	5000
PRJEB50289	ERR8054617	72785	55437	36400	29660	5000
PRJEB50289	ERR8054619	73234	56122	37026	36495	5000
PRJEB50289	ERR8054620	34067	23350	4628	3760	0
PRJEB50289	ERR8054717	56066	40932	12371	5740	5000
PRJEB50289	ERR8054719	75387	53448	21458	14675	5000
PRJEB50289	ERR8054720	39630	28430	13348	11109	5000
PRJEB50289	ERR8054721	95846	76986	63217	61129	5000
PRJEB50289	ERR8054722	110819	88193	66474	65877	5000

PRJEB50289	ERR8054724	34329	24227	9643	6320	5000
PRJEB50289	ERR8054725	131989	92610	45419	37416	5000
PRJEB50289	ERR8054726	80582	58594	27824	24746	5000
PRJEB50289	ERR8054727	28431	18503	7904	6765	5000
PRJEB50289	ERR8054729	112863	84356	22108	19418	5000
PRJEB50289	ERR8054730	135381	101155	51891	22276	5000
PRJEB50289	ERR8054731	106026	81391	49948	36120	5000
PRJEB50289	ERR8054732	53507	37315	18706	13429	5000
PRJEB50289	ERR8054735	124516	82047	6305	4052	5000
PRJEB50289	ERR8054736	24732	17831	6179	3554	5000
PRJEB50289	ERR8054737	108527	66195	14443	8286	5000
PRJEB50289	ERR8054738	105384	75191	4516	3661	0
PRJEB50289	ERR8054740	84509	54030	21805	20710	5000
PRJEB50289	ERR8054742	76958	55032	11614	9958	5000
PRJEB50289	ERR8054744	48334	31281	4514	3384	0
PRJEB50289	ERR8054745	12166	8929	3538	2003	0
PRJEB50289	ERR8054746	25735	18712	4210	3321	0
PRJEB50289	ERR8054747	48061	37395	11696	11004	5000
PRJEB50289	ERR8054748	8281	5347	1277	990	0
PRJEB50289	ERR8054749	39475	32895	25229	25122	5000
PRJEB50289	ERR8054750	34234	24085	8080	7104	5000
PRJEB50289	ERR8054751	14114	10611	3436	2464	0
PRJEB50289	ERR8054752	20552	14772	993	946	0
PRJEB50289	ERR8054753	59737	45352	29221	28466	5000
PRJEB50289	ERR8054754	89375	62208	6443	5735	5000
PRJEB50289	ERR8054756	116941	99627	96263	93469	5000
PRJEB50289	ERR8054971	42025	32503	23926	23367	5000
PRJEB50289	ERR8054973	59764	41172	13648	12526	5000
PRJEB50289	ERR8054974	47953	26222	5215	5129	5000
PRJNA347319	SRR4417507	44976	35237	33702	32610	5000
PRJNA347319	SRR4417508	73495	56752	54791	50647	5000
PRJNA347319	SRR4417509	462755	366962	366006	329410	5000
PRJNA347319	SRR4417510	159954	126289	125041	98477	5000
PRJNA347319	SRR4417511	572852	448681	448034	424905	5000
PRJNA347319	SRR4417512	381448	296323	295883	247650	5000
PRJNA347319	SRR4417513	416714	327468	326922	320487	5000
PRJNA347319	SRR4417514	571448	448608	446301	412743	5000
PRJNA347319	SRR4417515	209714	160470	159977	158953	5000
PRJNA347319	SRR4417516	106554	84371	84043	82107	5000
PRJNA347319	SRR4417517	556821	433650	432087	405999	5000
PRJNA347319	SRR4417518	417063	324114	322033	248159	5000

PRJNA347319	SRR4417519	17221	13380	13094	10546	5000
PRJNA347319	SRR4417520	6206	4954	4779	4444	0
PRJNA347319	SRR4417521	1946	1541	1440	1440	0
PRJNA347319	SRR4417522	412237	326488	324636	323246	5000
PRJNA347319	SRR4417523	32471	26180	26061	16834	5000
PRJNA347319	SRR4417524	23032	18641	18419	13178	5000
PRJNA347319	SRR4417525	54960	44345	44077	28636	5000
PRJNA347319	SRR4417526	27703	22301	22194	13135	5000
PRJNA347319	SRR4417527	28173	22501	22433	17540	5000
PRJNA347319	SRR4417528	432561	341413	340797	288053	5000
PRJNA347319	SRR4417529	524230	407434	406246	351617	5000
PRJNA412893	SRR6130729	120646	42131	40246	34213	5000
PRJNA412893	SRR6130730	321887	92661	87409	83395	5000
PRJNA412893	SRR6130731	566580	230251	194629	145518	5000
PRJNA412893	SRR6130732	411059	132310	111878	92382	5000
PRJNA412893	SRR6130733	529284	103106	85303	56405	5000
PRJNA412893	SRR6130734	435457	110821	100564	69589	5000
PRJNA412893	SRR6130735	188773	53178	35509	30493	5000
PRJNA412893	SRR6130736	96458	26255	16543	14636	5000
PRJNA412893	SRR6130737	236244	65128	57677	46157	5000
PRJNA412893	SRR6130738	296828	126195	110012	98835	5000
PRJNA412893	SRR6130739	155630	56019	51898	51844	5000
PRJNA412893	SRR6130740	215644	75335	63114	59316	5000
PRJNA412893	SRR6130741	139683	49611	44845	39823	5000
PRJNA412893	SRR6130742	319392	95101	83903	60821	5000
PRJNA412893	SRR6130743	371753	143869	127902	110449	5000
PRJNA412893	SRR6130744	134327	45887	44780	44496	5000
PRJNA412893	SRR6130745	308095	75317	67284	45737	5000
PRJNA412893	SRR6130746	271442	123852	101249	92074	5000
PRJNA412893	SRR6130747	293785	91424	73227	65277	5000
PRJNA412893	SRR6130748	210505	63904	52930	47432	5000
PRJNA412893	SRR6130749	139496	40361	28914	26597	5000
PRJNA412893	SRR6130750	241252	73207	64920	54461	5000
PRJNA412893	SRR6130751	220235	84541	74506	71425	5000
PRJNA412893	SRR6130752	218948	80804	69752	64152	5000
PRJNA412893	SRR6130753	394360	165523	136994	125247	5000
PRJNA412893	SRR6130754	175729	64590	57342	57034	5000
PRJNA412893	SRR6130755	526530	131014	110683	55786	5000
PRJNA412893	SRR6130756	655283	129500	105739	51686	5000
PRJNA412893	SRR6130757	438969	139591	123083	102757	5000
PRJNA412893	SRR6130758	538107	201508	168743	155468	5000

PRJNA412893	SRR6130759	486545	123556	97858	74351	5000
PRJNA412893	SRR6130760	472907	137467	117292	104963	5000
PRJNA412893	SRR6130761	389737	122950	97039	82579	5000
PRJNA412893	SRR6130762	552837	212825	177040	170570	5000
PRJNA412893	SRR6130763	468234	111768	89483	61177	5000
PRJNA412893	SRR6130764	505954	99893	87038	41797	5000
PRJNA719706	SRR14172012	62772	56655	53512	50850	5000
PRJNA719706	SRR14172015	66857	60661	58272	57447	5000
PRJNA719706	SRR14172016	41307	36915	35816	35595	5000
HI Dro	16S-10	82725	75172	73873	70639	5000
HI Dro	16S-100	80891	74455	72487	68822	5000
HI Dro	16S-101	125797	114196	112236	108065	5000
HI Dro	16S-116	57662	52018	51000	50004	5000
HI Dro	16S-118	86783	78531	76506	74081	5000
HI Dro	16S-120	150597	135824	132524	127082	5000
HI Dro	16S-122	91122	87242	85346	81122	5000
HI Dro	16S-134	111003	98378	96364	93261	5000
HI Dro	16S-135	142220	130018	126758	119389	5000
HI Dro	16S-137	137943	126063	124045	118443	5000
HI Dro	16S-138	115406	100856	98687	94819	5000
HI Dro	16S-139	182003	164343	160206	149490	5000

Table S6. *P-value* outcomes of pairwise beta-diversity comparisons at the genus level of bacterial (top) and fungal (bottom) community profiles shown in **Fig. S2**.

	Group 1[1]	Group 2	Beta-diversity[2]
**Multi-region 16S rRNA**	Europe	China + Japan	**0.006**
	Hawai'i	China + Japan	**0.002**
	Hawai'i	Europe	**0.001**
	UK	China + Japan	**0.001**
	UK	Europe	**0.001**
	UK	Hawai'i	**0.001**
	US_wild	China + Japan	**0.001**
	US_wild	Europe	**0.001**
	US_wild	Hawai'i	**0.001**
	US_wild	UK	**0.001**
	US_wild	US_lab	**0.001**
	US_lab	China + Japan	**0.001**
	US_lab	Europe	**0.001**
	US_lab	Hawai'i	**0.001**
	US_lab	UK	**0.001**
	HI_immigrans	China + Japan	**0.001**
	HI_immigrans	Europe	**0.001**
	HI_immigrans	US_wild	**0.001**
	HI_immigrans	UK	**0.001**
	HI_immigrans	HI_native	0.155
	HI_immigrans	Hawai'i	**0.001**
	HI_immigrans	US_lab	**0.001**
	HI_native	China + Japan	**0.001**
	HI_native	Europe	**0.001**
	HI_native	US_wild	**0.001**
	HI_native	UK	**0.001**
	HI_native	Hawai'i	**0.006**
	HI_native	US_lab	**0.001**
**Hawai'i populations 16S rRNA**	*D. immigrans*	Hawaiian *Drosophila*	0.160
	D. suzukii	Hawaiian *Drosophila*	**0.006**
	D. suzukii	*D. immigrans*	**0.003**
**Hawai'i populations ITS rRNA**	*D. immigrans*	Hawaiian *Drosophila*	0.162
	D. suzukii	Hawaiian *Drosophila*	0.081
	D. suzukii	*D. immigrans*	0.162

[1]Europe: Italy, France, Slovenia, Switzerland, and Spain; US_wild sites: New York and California; US_lab sites: New York and California; HI_immigrans: *D. immgrans* caught in Hawai'i; HI_native: native Hawaiian *Drosophilia.*
[2]Outcomes from PERMANOVA; *p*-values < 0.05 in bold.

Table S7. *P*-values for the outcomes of pairwise alpha- and beta-diversity comparisons at the family level of bacterial community profiles of lab populations shown in **Fig. S1**.

		Alpha-diversity[2]		
**Group 1[1]**	**Group 2**	**Chao1**	**Shannon**	**Beta-diversity[3]**
New York	California	0.055	**0.048**	**0.026**
China	California	0.055	**0.048**	**0.026**
China	New York	0.055	**0.048**	**0.026**

[1]The data set from Italy had only one sample and is excluded from statistical analyses.
[2]Outcomes from Wilcoxon Rank Sum tests; *p*-values < 0.05 in bold.
[3]Outcomes from PERMANOVA; *p*-values < 0.05 in bold.
